# Analysing Spatiotemporal Characteristics and Estimating the Spatial Distribution of Peste des Petits Ruminants (PPR) in Africa

**DOI:** 10.1155/tbed/9501187

**Published:** 2026-01-05

**Authors:** Rong Chai, Shuang Zhang, Dengata Lemu Joka

**Affiliations:** ^1^ College of Wildlife and Protected Area, Northeast Forestry University, Harbin, Heilongjiang Province, China, nefu.edu.cn; ^2^ Key Laboratory of Wildlife Diseases and Biosecurity Management of Heilongjiang Province, Harbin, Heilongjiang Province, China; ^3^ Sino-Ethiopian Wildlife Disease Research Joint Laboratory, Harbin, Heilongjiang Province, China

**Keywords:** Africa, maximum entropy (MaxEnt) model, Peste des petits ruminants (PPR), SDMs model, spatiotemporal analysis

## Abstract

Peste des petits ruminants (PPR) is a highly contagious disease that primarily affects small ruminants such as sheep and goats. Since first emerging in Africa, it has rapidly spread throughout the continent, causing significant mortality and posing a serious threat to livestock production and food security. In this study, we integrated diverse datasets using Geographic Information Systems (GIS) and employed the maximum entropy (MaxEnt) model to identify key drivers influencing the distribution of PPR outbreaks in Africa. Our comprehensive analysis provides critical insights into the spatial and temporal dynamics of PPR transmission, identifying shifting patterns of geographic spread, temporal clusters, and factors contributing to outbreak emergence and persistence over time. Based on existing research data, the results indicate a notable shift in the epidemic’s center of gravity from northwestern to southeastern Africa, offering strategic direction for future surveillance and control efforts. This study to understand and predict the distribution of PPR in Africa will help to develop a targeted surveillance program and analyze the trend of PPRV prevalence in Africa, which is important for the eradication and prevention of PPR.

## 1. Introduction

Peste des petits ruminants (PPR) is an acute, highly contagious viral disease that primarily affects sheep and goats, while also threatening small ruminant animals and vulnerable wildlife populations in many countries with outbreaks [1–3]. It can lead to morbidity and mortality rates as high as 90% among susceptible animals [4]. It is caused by the PPR virus (PPRV), which is a member of the genus *Morbillivirus* in the family *Paramyxoviridae* [5]. PPR exhibits various clinical symptoms, including fever, necrotizing stomatitis, gastroenteritis, pneumonia, and in severe cases, death [6]. The pathogenesis of PPRV infection leads to a marked weakening of the immune system, increasing vulnerability to secondary infections and making the clinical picture more complex [7]. PPRV shows affinity for epithelial (epitheliotropic) and lymphoid (lymphotropic) cells. Lymphoid and epithelial cell receptors like the signaling lymphocyte activation molecule (SLAM) and Nectin‐4. The respiratory tract is the main portal of entry; the virus is picked by the antigen presenting cells (APCs) and transported to the oropharynx, local lymphnode (pharyngeal and mandibular), and tonsils where replication occurs [8]. Diagnosis of the disease can be made based on clinical symptoms, pathological lesions, and the specific detection of viral antigens, antibodies, or genomes in clinical samples using various serological and molecular tests [9]. PPRV is closely related to other pathogenic viruses, such as rinderpest virus, measles virus, and canine distemper virus. The disease was first identified in Côte d’Ivoire in 1942 and has since spread to over 70 countries in Africa, the Middle East, and parts of Asia [10, 11]. PPRV transmission occurs through direct contact and respiratory droplets between infected and susceptible animals, with additional spread through ingestion of contaminated feed or water [12, 13, 14]. The disease is particularly prevalent in regions with high populations of small ruminants, making it a major concern for farmers in endemic areas [15, 16]. It poses a significant economic threat to livestock farmers, especially in regions where small ruminants are the primary source of livelihood [17, 18]. Moreover, PPR’s impact on Africa extends beyond animal health, with significant implications for economic stability and trade dynamics in the region. Several studies highlight the critical role of the disease’s prevalence and the challenges it poses to small ruminant farmers, which are crucial to the local economy. Samuel E. Mantip emphasizes that since the disease was discovered, PPR has had a major impact on sheep and goat farmers in Africa [19], leading to extensive research on virus isolation, molecular epidemiology, and vaccine development [20]. PPR outbreaks have caused severe economic impacts on farmers, with losses resulting from mortality being the primary source of such losses [21]. The effect of PPR on this small ruminant population is substantial, with significant implications for Africa.

All evidence to date suggests that PPR originated in Africa [22] and has been spreading across parts of sub‐Saharan Africa since 1993, continuing for several decades [23]. Subsequently, in East Africa, serological evidence of PPR was found in Uganda, Sudan, Tanzania, and Ethiopia, indicating the spread of the disease in East Africa [24–28].The study by Luka et al. provided crucial evidence for the presence of PPRV in the region through a combination of serological and molecular methods [29]. In 2008, Morocco experienced its first PPR outbreak [30], with the highland goat breed, common in both Morocco and Europe, found to be highly susceptible to PPRV, exhibiting severe symptoms and a high mortality rate of up to 85% in young animals [13, 31]. Between 2010 and 2016, widespread cross‐border transmission occurred in West Africa, with outbreaks reported in Senegal, Guinea, and Mali [30, 32]. In 2015, PPR outbreaks occurred in Cheraga in northern Algiers and in the northwest of Morocco, potentially posing a threat to Europe [33]. In 2017, an outbreak occurred in the North Kivu province of the Democratic Republic of Congo (DRC), resulting in significant sheep and goat mortality [33, 34]. Between 2022 and 2023, Mali experienced large‐scale outbreaks despite conducting annual vaccination campaigns, though coverage remained low [35]. In 2024, the World Organization for Animal Health (WOAH) reported an outbreak, with laboratory tests showing that 37 out of 58 sheep tested had fallen ill and died, with a mortality rate of 63.8%. Today, PPR is widely spread across Africa, primarily active in the North and Central African countries, causing significant impacts on the local economy and food security.

The PPR outbreak is not only an animal health issue but has also become an important public health event impacting regional economic stability and the security of international animal trade [36]. Under the framework of the eradication program jointly promoted by the Food and Agriculture Organization (FAO) and the WOAH, PPR is listed as the animal disease most likely to be globally eradicated after rinderpest. However, achieving the global eradication target by 2030 still faces key challenges, such as unclear identification of high‐risk areas, ambiguous cross‐border transmission routes, and insufficient capacity for accurate risk prediction.

Environmental and climatic factors drive PPR transmission. Social network analysis reveals that Tanzanian pastoralists migrate en masse during the dry season due to pasture degradation, leading to increased animal contact frequency. This results in a 2.3‐fold higher risk of PPR transmission compared to the rainy season [37].

The small ruminant industry in Africa is highly intertwined with livelihoods. Cross‐border grazing and trade in livestock products form a network characterized by high mobility and high contact, making the “hotspot‐corridor‐barrier” spatial pattern particularly prominent. Meanwhile, Africa spans multiple Köppen climate zones, with complete gradients in climate, vegetation, and land cover. This facilitates hierarchical modeling within the same continent, helping to control ecological heterogeneity and enhance interpretability. More importantly, multiple countries have maintained continuous reporting to WOAH/WAHIS and FAO EMPRES‐i; when combined with Supporting information from the literature, these data can support report‐based spatial risk assessments and transparent discussions on reporting biases. Therefore, selecting Africa not only holds significant implications for public health and development but also provides an ideal testbed for establishing a replicable methodological framework of “zoned species distribution models (SDM) + spatial cross‐validation.“

This study aims to demonstrate the trajectory of PPR epidemic patterns and the mechanisms underlying its clustered diffusion, thereby enhancing the understanding of the spatial and temporal dynamics of PPR outbreaks. To achieve this, spatiotemporal analysis methods such as hotspot analysis, kernel density analysis, standard deviation ellipse (SDE) analysis, and spatial autocorrelation analysis were used to examine the spatial distribution characteristics, transmission directions, and spatial clustering patterns of PPR in Africa. Due to the varying climatic conditions supporting species distribution and biodiversity gradients in different regions, we divided the African region into subregions based on the Köppen climate classification map for distribution prediction. Using the maximum entropy (MaxEnt) model in combination with ecological, geographic, and meteorological factors, a risk zone prediction model for PPR outbreaks in Africa was established, identifying risk factors associated with PPR outbreaks and providing important evidence for prevention and control decision‐making.

## 2. Materials and Methods

### 2.1. Outbreak and Case Data of PPR in Africa

Information on PPR across Africa was obtained from the FAO (http://www.fao.org/home/en/), the WOAH (https://www.woah.org/en/home/), the Global Animal Disease Information System (http://www.example.com = 0, EMPRES‐i) and relevant literature on PPR outbreaks and epidemiological data [38]. The outbreak data primarily came from diagnostic testing by national laboratories and were reported to WOAH and FAO. The information mainly includes the specific time of the outbreak, the geographical location of the outbreak points, and the number of reported cases. To ensure the accuracy of the data from different sources, duplicate coordinates and incomplete information were removed.

### 2.2. Preliminary Screening of Environmental Variables

We selected five types of environmental variables to construct the SDM model: climate variables, topographic variables, anthropogenic impact variables, soil variables, and vegetation variables. The corresponding data sources were: CHELSA 1.2 for meteorological data, including 67 variables; the US Geological Survey 90‐meter Digital Elevation Model (DEM) for topographic data; WorldPop for population density; the Climate Change Initiative (CCI) datasets for vegetation and land cover; and the FAO Soil Portal for soil data. All spatial data were preprocessed and calculated using standard operations in ArcGIS 10.8, and projected in UTM‐WGS‐1984. All environmental variable data were resampled to 30 arc‐seconds (~1 km^2^ on the ground). Table 1 shows the layers, sources, categories, and variables used in the MaxEnt model for environmental predictive variables.

**Table 1:** The environmental predictor variables utilized in MaxEnt modeling, including data layers, sources, categories, and variables/proxy.

Layers	Source	Value/categories	Variable/proxy
Climate^a^			
Monthly P (prec1‐12)	CHELSA	0 to 1201 mm/month	Precipitation
Monthly mean T (temp1‐12)	CHELSA	−54.9 to 39.2°C	Mean temperature
Monthly min T (min1‐12)	CHELSA	−56.5 to 32.3°C	Minimum temperature
Monthly max T (max1‐12)	CHELSA	−53.2 to 47.3°C	Maximum temperature
Bioclimatic (bio1‐19)	CHELSA		Annual trends, seasonality, extreme or limiting environmental variables
Terrain			
Elevation^b^	ASTER‐GDEM	−328 to 4739 ma.s.l	Climbing distance
Human impact			
Human population^c^	WorldPop	0 to 1202.6 ind/km^2^	Human–Animal interaction
Vegetation			
Land cover^d^	ESA	Cropland (3), herbaceous, tree (9), shrubland (3), grassland, urban areas, bare areas (2), mosaic shrub and herbaceous cover, water bodies, permanent snow, and ice	Human activity venues
Soil			
World soil databas^e^	IIASA		Soil type

Abbreviations: P, precipitation; T, temperature.

^a^Source: http://chelsa-climate.org/.

^b^Source: http://www.gscloud.cn/.

^c^Source: https://www.worldpop.org/.

^d^Source: https://maps.elie.ucl.ac.be/CCI/viewer/.

^e^Source: https://www.fao.org/soils-portal/data-hub/soil-maps-and-databases/faounesco-soil-map-of-the-world/en/.

### 2.3. Regional Division Strategy

The Köppen climate classification system is widely used due to its clear boundaries, straightforward application, and greater suitability for represented landscape zones compared to other classification methods. Studies have shown that climate influences the incidence and distribution of infectious diseases. The Köppen–Geiger global 1‑km climate classification maps were downloaded from https://www.gloh2o.org/koppen/. The Köppen climate classification divides the global climate into five major categories based on latitude, from the equator to the poles: A (tropical climate), B (dry climate), C (subtropical climate), D (temperate and subarctic climate), and E (polar climate). Each major category is further subdivided into several minor types. A total of 31 minor types are defined globally, each represented by two or three letters. The main climate types in Africa are A, B, and C zones.

## 3. Methods

### 3.1. Geographic Information Systems (GIS)‐Based Analytical Methods

**Figure 2:**
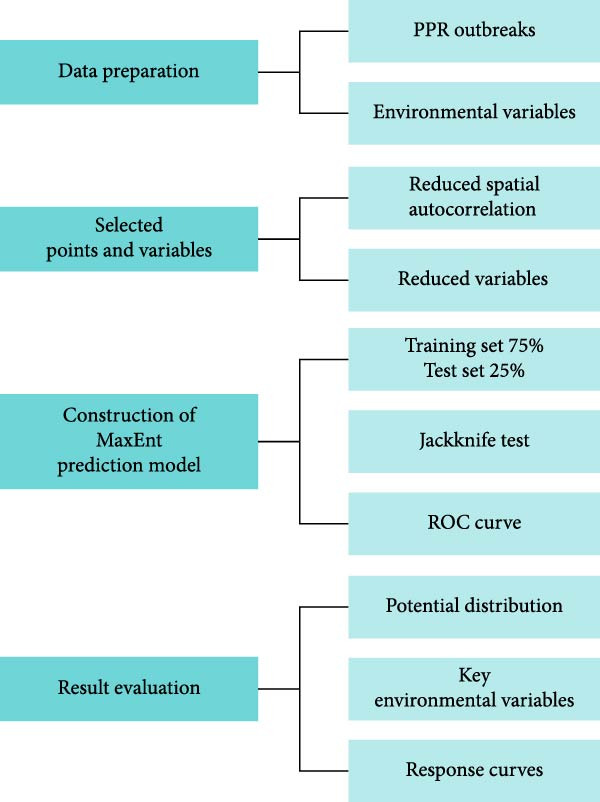
Flow chart of MaxEnt model construction.

Clarke et al. proposed three types of spatial analysis models for animal diseases: visualization, exploratory data analysis, and model construction [39]. Among these three methods, spatial visualization techniques integrate GIS with epidemic data, enabling the direct and accurate illustration of the distribution patterns of PPR in Africa. The second method is exploratory data analysis, which identifies specific spatial patterns by filtering spatial data. Given that infectious diseases exhibit both spatial and temporal dynamics [40], static exploratory analysis methods for disease patterns are often inadequate for capturing their complex spatiotemporal behavior. Therefore, spatial autocorrelation methods and spatiotemporal scan analysis are required to explore the spatiotemporal patterns of disease transmission [41]. Model construction is the final method for spatial analysis. We employ GIS to process the required data for model input, simulate spatial processes, and present results through maps. To reveal the spatial distribution and transmission trends of the PPR epidemic in Africa, this study utilizes GIS technology for four types of spatial analysis: ① Hotspot analysis to identify clustered high‐incidence areas; ② Kernel density estimation to characterize high‐density outbreak zones; ③ SDE to describe the spatial dispersion characteristics and main directional trends of the outbreak regions; ④ Linear directional mean (LDM) analysis to study the virus transmission paths. Figure 1 presents the analytical flowchart for the PPR epidemic in Africa, and Figure 2 outlines the technical route for MaxEnt in this study.

Figure 1The overall workflow of this study. (a) Data preparation and model setting, (b) PPR outbreaks and distribution, (c) spatial propagation trend, and (d) MaxEnt model results.

**Figure:**
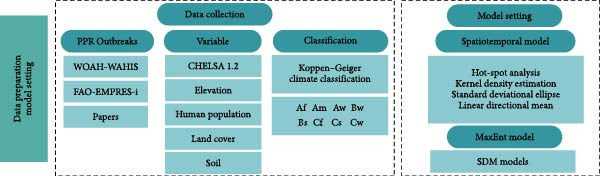
(a)

**Figure:**
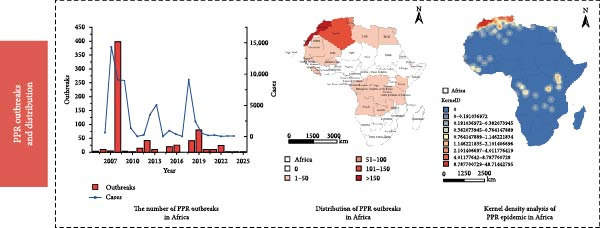
(b)

**Figure:**
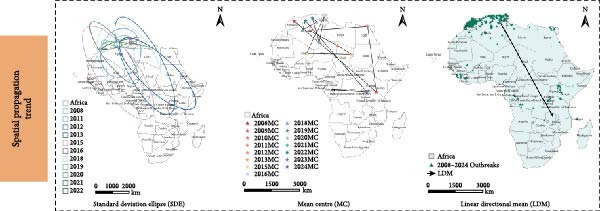
(c)

**Figure:**
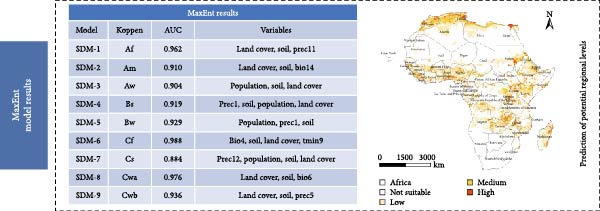
(d)

#### 3.1.1. Hotspot Analysis

Hotspot analysis can accurately identify high‐value clusters (hotspots) and low‐value clusters (cold spots) that are statistically significant. Its core function lies in detecting clustering or dispersion patterns in spatial distributions. This method has been widely applied in areas such as animal disease trend analysis, medical health service demand assessment, and soil heavy metal pollution analysis.

The Getis‐Ord Gi^∗^ index is a widely used local spatial statistic for identifying statistically significant clusters of high (hot spots) and low (cold spots) values within a defined geographic area. In this analysis, the spatial relationship parameter was defined using the fixed‐distance band conceptualization, employing the Euclidean distance method for calculating spatial proximity. All other parameters were maintained at their default settings in ArcGIS software. This approach facilitates the detection and characterization of spatial clustering patterns relevant to the study objectives.

#### 3.1.2. Kernel Density Estimation

Kernel density estimation allows for a comprehensive analysis of data clustering within a specific area and its radiative impact on surrounding regions. The results of this analysis provide a solid theoretical and data‐driven foundation for identifying high‐risk areas for diseases. From a technical perspective, kernel density analysis uses a kernel function to calculate the value per unit area based on input point or polyline features. Each point or polyline is then converted into a smooth conical surface, thereby displaying the spatial distribution characteristics of the data.

In this study, we used outbreak data collected from PPR sampling points as the base data for kernel density estimation. During the specific analysis process, the parameters were set using the system’s default values. This approach not only follows conventional analysis standards but also ensures the stability and comparability of the results, thus more accurately revealing the underlying spatial distribution patterns and providing valuable reference information for subsequent disease prevention and control efforts.

#### 3.1.3. SDE

The SDE, as a widely used analytical tool, allows for an in‐depth exploration of the overall spatial characteristics of geographic features from both a global perspective and spatial dimension. In the field of animal disease epidemiology, SDE has a wide range of applications: it can be used not only for dynamic disease monitoring to grasp the spread of disease in real‐time but also to support the study of healthcare service utilization and provide a basis for optimizing the allocation of medical resources.

SDE includes several common basic parameters: the center of the ellipse, the long axis, the short axis, and the azimuth angle. Each of these parameters carries specific information: the spatial range covered by the ellipse represents the main distribution area of the geographic features, clearly showing the primary distribution range; the center of the ellipse marks the central location of the entire ellipse, serving as the core reference point for the distribution; the azimuth angle visually reflects the main directional trend of the geographic feature distribution, allowing us to understand the extension direction of the features in space; the long axis reflects the degree of dispersion of the features along the main trend direction, with a longer axis indicating greater dispersion in that direction. The establishment of the SDE requires more than three non‐coincident points, so data from years that do not meet this requirement are excluded.

In this study, during the SDE analysis using ArcGIS software, the size of the ellipse was set to cover 68% of the outbreak points. This setting ensures the accuracy of the analysis while better fitting the actual needs of the study, specifically investigating the distribution characteristics of disease outbreak points and providing a reliable spatial distribution reference for subsequent related analyses.

#### 3.1.4. Mean Center (MC) and LDM

MC Basic Logic: The MC is calculated by finding the weighted geometric center of a set of spatial points, reflecting the concentration trend of PPR outbreaks in both spatial and temporal dimensions. In the MC map, the black connecting lines can be viewed as the spatial correlation of PPR epidemics (epidemic transmission chains). By analyzing the direction of these connecting lines, we can extract patterns of epidemic spread direction.

LDM is a spatial analytical method used to quantify the average direction or azimuth of a set of line features. Its main function is to calculate the average direction or azimuth of a set of polylines. Due to this characteristic, LDM can be effectively applied in analyzing the direction of epidemic transmission, providing strong support for studying the disease’s diffusion paths. In terms of parameter characteristics, the length of LDM corresponds to the average length of all polyline elements, while its final direction is consistent with the average direction of all polyline vectors. These parameters together form the core elements of LDM analysis.

In this study, to analyze the development trend of the PPR epidemic in Africa, we used PPR sampling data (outbreak data) from 2008 to 2024 and applied LDM for related calculations.

For specific operational details, please refer to the Supporting Information. Through this series of operations, we are able to more accurately grasp the transmission trend of the PPR epidemic in Africa and provide scientific evidence for the development of epidemic control strategies.

### 3.2. MaxEnt‐Based Prediction of PPR Epidemics

We have specifically clarified in the revised manuscript that this study evaluates the “reporting‐based outbreak risk of PPR outbreaks as reported in the FAO WOAH‐WAHIS EMPRESSi database,” rather than the absolute distribution of “true prevalence. By using SDM v1.1c in ArcGIS 10.8, all recorded PPR locations were filtered to minimize spatial autocorrelation [42, 43]. The filtering was performed by restricting the minimum distance between each pair of points, which is considered the most effective method to correct sampling bias [44]. We used natural break settings with a maximum distance of 50 km and a minimum distance of 5 km [43]. Principal component analysis (PCA) was performed using SPSS 22.0 to evaluate 67 environmental variables. This analysis reduced the dimensionality of the dataset by identifying the principal components that explain the majority of the variance, from which the primary climate predictor factors were selected [45]. We extracted environmental values behind the distribution points in ArcGIS 10.8, and used eigenvalues greater than 1.0 and the scree plot standard or the “elbow” stopping rule in the PCA factor decomposition at the item level. Unnecessary loading and rotation of climate variable factor patterns were applied to retain the predictors for subsequent analysis in MaxEnt. Subsequently, the filtered PPR occurrence locations and selected predictor factors were used as input data for the MaxEnt model [46]. The area under the curve (AUC) of the receiver operating characteristic (ROC) curve was selected to ensure the robustness of the MaxEnt model. The selected presence records were divided into 75% for training and 25% for testing, based on 10 bootstrap repetitions to build and validate the model. For the remaining parameters, default settings from the pilot study were retained. Additionally, jackknife tests and variable response curves were used to determine the relative contribution of predictive variables to the model. Stepwise elimination was applied to remove multicollinear predictors and exclude those contributing less than 10% to the explained variance, streamlining the model. And variables with high standard deviation, indicating unstable responses, were also removed based on visual inspection, ensuring a robust and biologically meaningful predictor set [47].

Finally, variance inflation factor (VIF) analysis was conducted to assess the multicollinearity among the reduced predictor factors [48, 49]. VIF values below 10 indicate low and acceptable multicollinearity [50]. For visualization, the model risk map was divided into four risk categories: high, medium, low, and very low, using the Jenks natural breaks optimization method to identify high‐risk areas.

## 4. Results

### 4.1. Spatiotemporal Distribution Characteristics

#### 4.1.1. Annual Epidemic Changes and Trends

Supporting Information: Table S1 presents the annual outbreak data of PPR across several African countries from 2005 to 2025, clearly illustrating the complex evolution of the epidemic. Between 2005 and 2014, most countries experienced a silent epidemic phase, with no outbreaks recorded annually. However, in 2008, Morocco and Zambia each experienced peaks of 397 and 398 outbreaks, respectively, becoming prominent “epidemic hotspots” during this phase. Later on, countries such as Algeria and the DRC began reporting sporadic cases, indicating a gradual spatial expansion of the disease. Nevertheless, the overall scale was small and the affected countries were limited, with the epidemic exhibiting a localized sporadic pattern. From 2015 to 2025, the epidemic pattern changed dramatically, with a significant expansion in the spread of the disease. Countries that previously had no cases, such as Angola (2015) and Burkina Faso (2017), began reporting cases, indicating an increased risk of PPR spread. The virus may have crossed geographical barriers through animal migration and trade activities.

Figure 3 presents the PPR epidemic data in Africa, revealing the following patterns from both temporal and spatial dimensions:

Figure 3Line chart of peste des petits ruminants outbreak data and its geographical distribution in Africa. Figure (a), the red bar chart represents outbreaks of Peste des Petits Ruminants (PPR), while the blue curve indicates the number of PPR cases. Figure (b) white indicates zero cases, with darker shades representing a higher number of outbreaks.

**Figure:**
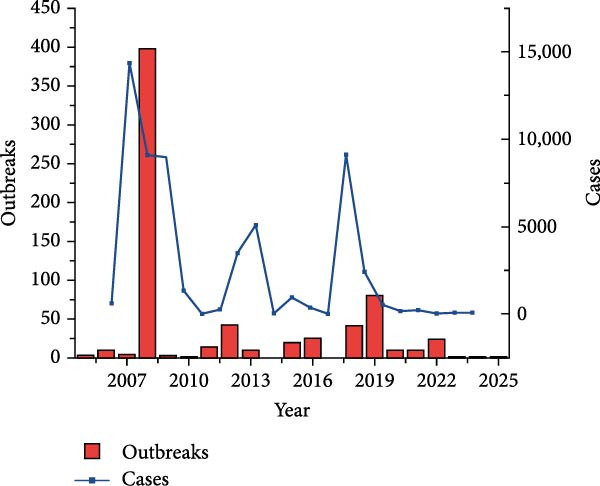
(a)

**Figure:**
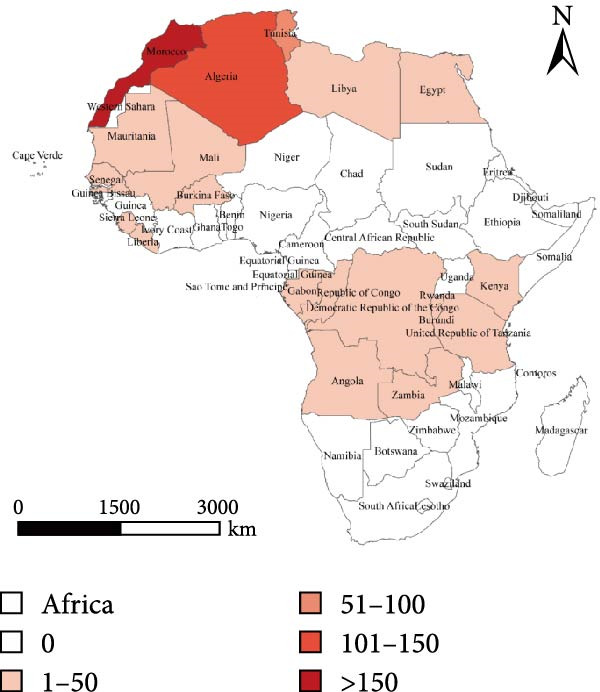
(b)

Temporal Dimension: The number of outbreaks peaked between 2008 and 2009, indicating that the epidemic was concentrated and highly active during this phase. After fluctuating and declining from 2010 to 2015, there was a slight rebound, suggesting periodic fluctuations in the epidemic. In 2017, the number of cases (blue line) spiked, leading to an increase in outbreaks. Although the numbers later decreased, fluctuations continued, indicating that the epidemic had not fully subsided. From 2021 to 2025, there was an overall downward trend, reflecting the gradual easing of the epidemic. The trends in case numbers and outbreaks are highly correlated; during peak periods, both surged simultaneously, highlighting the transmission dynamic whereby a higher number of outbreaks leads to more infections. The subsequent decline corresponds with the epidemic being largely controlled.

Spatial Dimension: West Africa is the epicenter of the epidemic. These regions may experience frequent animal trade, high and dispersed livestock density, and a climate conducive to viral transmission, making them hotspots for the epidemic. In the map, lighter and white regions (such as parts of southern Africa) show areas with lighter or no outbreaks, as natural barriers (geographic isolation, deserts) prevent transmission, forming “buffer zones” for the epidemic.

Comprehensive Pattern: In years of high epidemic peaks, high‐risk areas may expand in range, reflecting the virus’s spatiotemporal spread—first emerging in trade and livestock‐intensive areas, then infiltrating surrounding regions through animal movements and human interaction, intensifying the regional epidemic pressure.

##### 4.1.1.1. Geographic Clustering and Hotspot–Coldspot Distribution

Figure 4a shows the hotspot–coldspot analysis of the PPR outbreaks in Africa. The analysis reflects the number of cases in each outbreak event. The hotspot areas are mainly concentrated in East Africa: countries in East Africa (such as Ethiopia and surrounding areas of Kenya) exhibit high‐confidence hotspots (95%–99%), indicating that although the frequency of outbreaks is low in this region, their scale is large. The coldspot areas are concentrated in the northern part of West Africa: Northern West Africa (such as areas near Mauritania and Morocco) shows high‐confidence coldspots (95%–99%), indicating frequent outbreaks in this region, but with a small scale.

Figure 4Hotspot analysis of PPR in Africa. (a) Cold and hot‐spot analysis of PPR epidemic in Africa. (b) Kernel density analysis of PPR epidemic in Africa. The data of PPR outbreak in Africa were collected from WOAH (https://www.woah.org/en/home/) and EMPRES‐i (http://empres-i.fao.org/eipws3g/#h=0) and the map was created by ArcGIS software (version 10.8).

**Figure:**
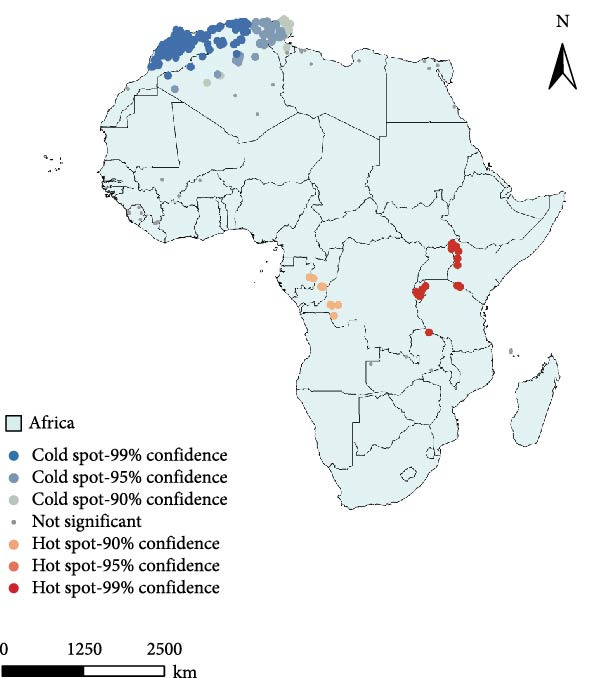
(a)

**Figure:**
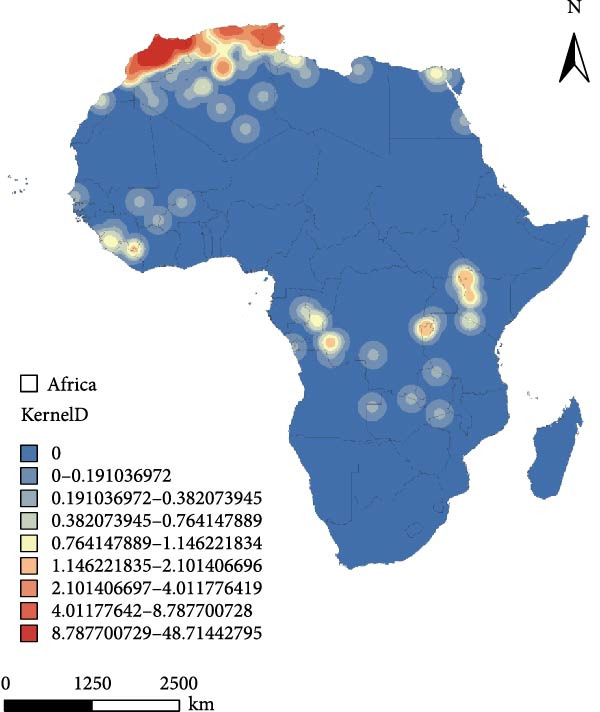
(b)

Figure 4b shows the kernel density analysis of the PPR outbreaks in Africa. The coastal regions of Northern West Africa, including Morocco and Algeria, display significant red and orange high‐density areas, indicating that these are the core hotspots where PPR outbreaks are most concentrated and frequent. In East African countries, such as Kenya and Ethiopia show yellow‐orange patches, which represent secondary high‐density areas, reflecting the regional clustering of the disease in East Africa. The yellow and light blue patches in West, Central, and East Africa represent areas with moderate outbreak density, which act as buffer zones for disease spread. While the disease has not experienced large‐scale outbreaks in these regions, there is still a risk of transmission. Large blue areas in Central and Southern Africa, show low kernel density values. The analysis results show that the highest outbreak density value is 48.71 outbreaks per square kilometer. The high‐incidence areas for PPR in Africa are mainly along the coastal regions of North Africa, including Morocco and Algeria. Areas with high spatial kernel density have a high likelihood of PPR outbreaks.

### 4.2. Transmission Path and Directional Analysis

#### 4.2.1. MC and SDE Analysis

To examine the spatiotemporal dynamics of PPR outbreaks in Africa, Figure 5e presents the MC map for several years. For each year, the MC indicates the spatial centroid of outbreak occurrences, highlighting the core areas of epidemic concentration and revealing shifts in the primary distribution and migration patterns of the disease over time.

Figure 5Spatial propagation trend in Africa from 2008 to 2024. (a) Standard deviation ellipse (SDE) of PPR outbreak from 2008 to 2013. (b) Standard deviation ellipse (SDE) of PPR outbreak from 2015 to 2016. (c) Standard deviation ellipse (SDE) of PPR outbreak from 2018 to 2022. (d) Standard deviation ellipse (SDE) of PPR outbreak from 2008 to 2022. (e) Mean center (MC) of PPR outbreak from 2008 to 2024. (f) Linear directional mean (LDM) of PPR outbreak in Africa from 2008 to 2024. The arrow indicates the direction of PPR epidemic in Africa. The data of PPR outbreak in Africa were collected from WOAH (https://www.woah.org/en/home/) and EMPRES‐i (http://empres-i.fao.org/eipws3g/#h=0) and the map was created by ArcGIS software (version 10.8).

**Figure:**
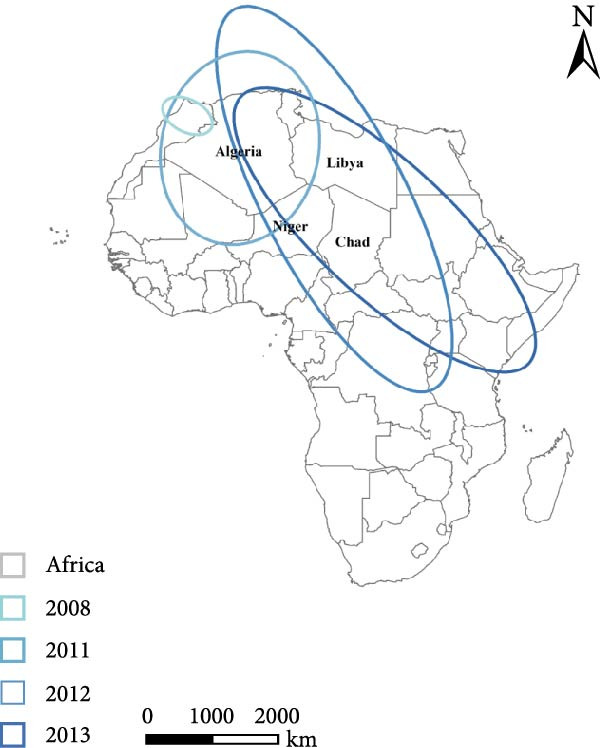
(a)

**Figure:**
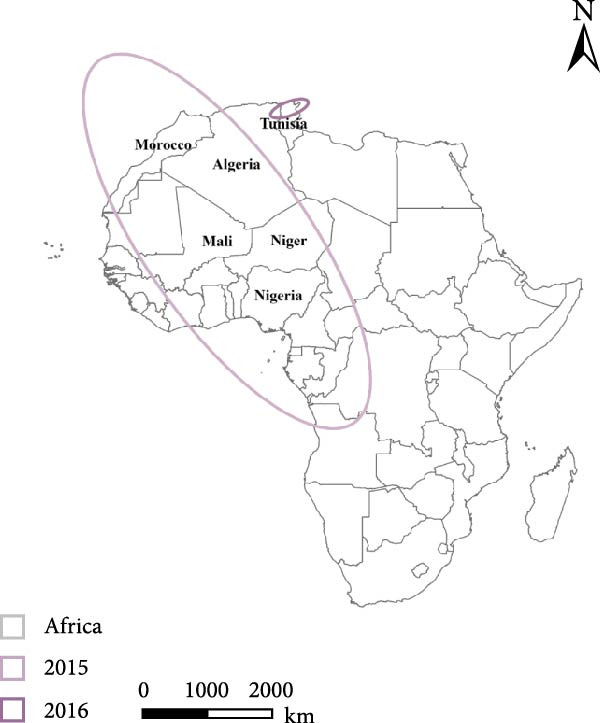
(b)

**Figure:**
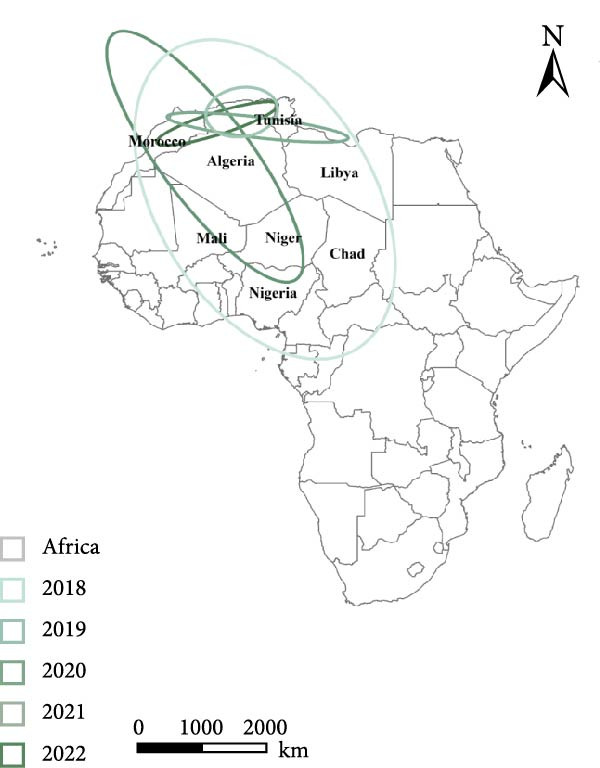
(c)

**Figure:**
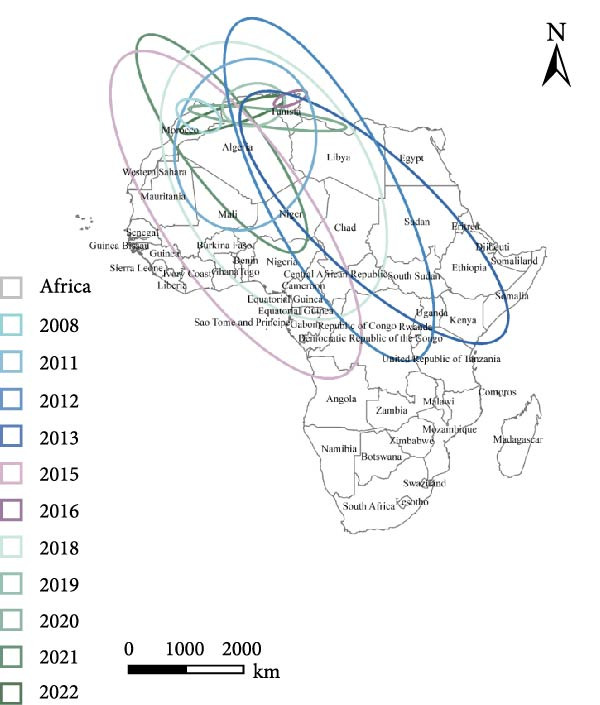
(d)

**Figure:**
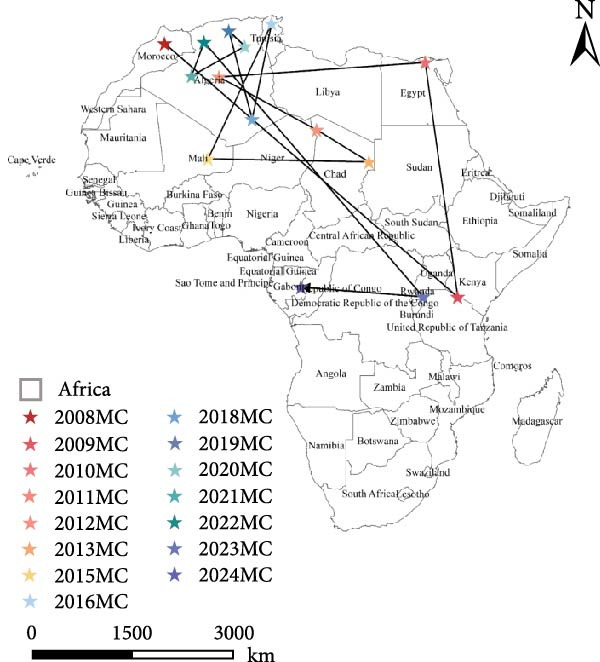
(e)

**Figure:**
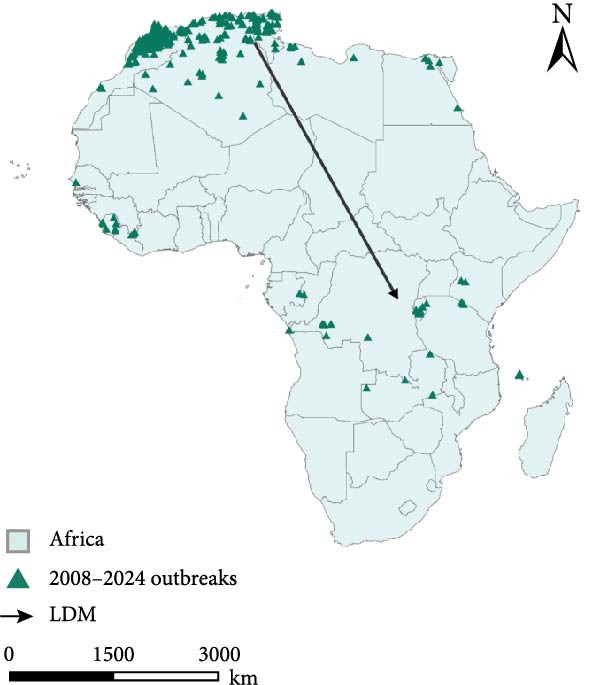
(f)

From 2008–2024, the overall dominant transmission direction of PPR in Africa was from the northwest to the southeast, with some localized radiations. From the lines shown in the figure, the connections between North Africa, East Africa, and West Africa generally follow a “northwest → southeast” direction, reflecting the cross‐regional spread of PPR from the northwest of Africa to the southeast.

There were temporal variations in the PPR outbreaks in Africa from 2008 to 2024. In the early stages, the outbreaks were concentrated in the northwest, while in the later stages, they extended southeast. From 2008 to 2015, the monitoring points were primarily concentrated in North Africa (Morocco, Algeria), and the outbreak spread was mainly within the northwest region (Morocco → Algeria), with a “short‐distance, localized” characteristic. From 2016 to 2024, the monitoring points expanded southeastward (Kenya, Congo, etc.), and the outbreak lines crossed larger geographical area, with a significant extension toward the southeast (Algeria → Kenya, Mali → South Sudan). This indicates that PPR spread toward Central and Eastern Africa over time, expanding from isolated “point” outbreaks to a broader “cross‐regional band” of transmission.

The high frequency transmission lines in the figure are concentrated along the corridor from Morocco to Mali and onward to Kenya forming the main axis of PPR transmission in recent years. This axis coincides closely with livestock migration routes and live animal trade corridors in Africa (such as the Sahel region’s pastoral transitions and cross‐border trade between North and East Africa). This indicates that the spread of PPR is driven by human activities, following economic cooperation networks. The “North Africa, West Africa → East Africa, Southern Africa” corridor has been the key transmission route for the spread of PPR in Africa from 2008 to 2024.

Figure 5a shows the SDE based on PPR outbreak data in different years across Africa. From 2008 to 2013, the SDE of PPR outbreaks gradually expanded, with an increasing degree of dispersion and a trend toward stabilization in the direction of spread. This reflects the gradual shift of PPR outbreaks in Africa from localized, small‐scale clusters to a broader regional spread along a specific direction (such as northeast–southwest). From 2015 to 2016, Figure 5b shows that the SDE expanded further, with the semiaxis growing, clearly indicating the spatial diffusion characteristics of PPR outbreaks: sustained spread along the northeast‐southwest direction, which is associated with animal migration and trade activitie. From 2018 to 2022, Figure 5c shows that the SDE of PPR outbreaks in Africa progressively expanded along the northeast‐southwest axis, with increasing spatial dispersion each year. This expansion extended the affected area from North and West Africa into Central Africa, reflecting an elevated risk of cross‐regional transmission and broader epidemic spread Figure 5d.

#### 4.2.2. Linear Direction and Propagation Channel Identification

LDM map (Figure 5f) is describing the transmission trend of PPR outbreaks in Africa. The LDM calculates the dominant direction of diffusion for a set of spatial points, quantifying the “main transmission path” of the PPR epidemic center. The black arrows LDM in the figure represent the average direction of spread, based on PPR outbreak points (green triangles) from 2008 to 2024, revealing the spatiotemporal trend of epidemic diffusion.

The key transmission corridor for PPR epidemics in Africa between 2008 and 2024 spans from North Africa and West Africa to East and Southern Africa. Along the LDM arrow path, the PPR outbreak points show a gradient distribution:

North African origin zone (Morocco, Algeria): Outbreak points are densely concentrated here, marking the early epicenter of the PPR epidemic where the virus initially gathered and began to spread.

West African transition zone (Mali, Niger): Outbreak points extend along the arrow path, acting as an intermediary corridor for the northwest‐to‐southeast transmission, accelerating the virus’s movement across regions.

East African diffusion zone (Kenya, Tanzania): Outbreak points deepen into East Africa along the transmission corridor, leveraging the region’s dense local livestock trade network—including cross‐border livestock transportation—to facilitate and expand the epidemic’s reach.

Southern African terminal zone (Malawi, Mozambique): The outbreak points at the end of the arrow mark the spread of PPR into Southeast Africa, forming a complete transmission chain from the northwest origin to the southeast diffusion.

### 4.3. Model Construction and the Impact of Meteorological Factors on the Model

**SDM-1**: After filtering, a total of 8 PPR outbreak points were obtained, each at least 5 km apart. The MaxEnt model achieved an ROC curve AUC of 0.962, indicating excellent predictive accuracy and strong model performance in discriminating suitable conditions for PPR outbreaks from background locations.. With the land cover type 190 (Urban areas) and the soil type 4 (LITHOSOLS), the highest probability of PPR presence occurs during November precipitation (Prec11) within the 50–100 mm/month range.

**SDM-2**: The probability of PPR presence was highest when the land cover type was 190 (Urban areas) and the soil type was 115 (Orthic Ferralsols). The precipitation in the driest month (Bio14) indicated that as precipitation increased, the probability of PPR presence gradually decreased.

**SDM-3**: The probability of PPR presence was also significantly influenced when the soil types were 34 (Humic Gleysols) and 32 (Haplic Xerosols), and when the land cover types were 20 (Irrigated cropland), 210 (Water bodies), and 120 (Shrubland).

**SDM-4**: As January precipitation increased, the probability of PPR presence first increased and then decreased, reaching its peak of about 0.78 when precipitation was ~40 mm/month. The probability of PPR presence was highest when the soil type was 79 (Gleyic Solonchaks) and the land cover type was 60 (Deciduous broadleaved). The probability of PPR presence sharply increased when population density exceeded 0 persons/km^2^ and continued to increase monotonically with higher population density.

**SDM-5**: The probability of PPR presence sharply increased when population density was greater than 0 persons/km^2^ and then stabilized between 0.8 and 0.9. The probability of PPR presence was highest when the January precipitation (Prec1) ranged from 20 to 40 mm/month. The Model show that soil type 90 (Chromic Vertisols) also had a significant impact on the probability of PPR presence.

**SDM-6**: The seasonal variation in temperature reflects the average temperature and its variation range. The greater the amplitude of seasonal temperature variation, the lower the probability of PPR presence. The probability of PPR presence was also significantly influenced by soil type 3 (Gelic Planosols), land cover types 100 (Mosaic herbaceous cover/tree and shrub). As the minimum temperature in September (Tmin9) gradually increased, the probability of PPR presence also increased. When the minimum temperature in September was between 18°C and 20°C, the probability reached its maximum.

**SDM-7**: As the precipitation in December (Prec12) gradually increased, the probability of PPR presence first increased and then decreased. The probability reached its optimum when the precipitation was in the 50–100 mm/month range. The probability sharply increased when the population density was greater than 0 persons/km^2^ and then gradually decreased as the population density further increased. The probability of PPR presence was also significantly influenced by soil type 18 (Eutric Cambisols) and land cover types 190 (Urban areas), 100 (Mosaic tree and shrub/herbaceous cover), and 11 (Rainfed cropland), with probabilities greater than 0.8.

**SDM-8**: Land cover type 40 (Mosaic natural vegetation), soil types 115 (Orthic Ferralsols) and 112 (Rhodic Ferralsols) had significant impacts on the probability of PPR presence. The response curve for the lowest temperature in the coldest month (Bio6) indicated that as the minimum temperature in the coldest month increased, the probability of PPR presence also increased. The probability reached its optimum when the minimum temperature in the coldest month was around 15°C.

**SDM-9**: The probability of PPR presence was significantly influenced by land cover type 190 (Urban areas) and soil type 52 (Cambic Arenosols). The response curve for May precipitation (Prec5) indicates that as precipitation increases, the probability of PPR presence gradually decreases, with the highest probability observed when May precipitation ranges from 0 to 50 mm/month.

The contents of the appendix figure mainly reflect the model accuracy and important influencing factors of SDM1−9. The area under the ROC curve (AUC) is a threshold independent criterion. It was embedded in the MaxEnt and was used to assess the goodness‐of‐fit of the model. A larger AUC value indicates a higher correlation between environmental variables and the predictive model, and the accuracy of the model’s prediction. The evaluation criteria are: 0.7–0.8, which means the prediction accuracy is good, 0.8–0.9, which means that the prediction accuracy is relatively good, and 0.9–1.0, indicates that the prediction accuracy is very good. ROC curves for SDM1−9 are plotted in Appendix figure. Response curves for environmental factors are plotted in Appendix Figures 2 and 3, which show the mean response (red) and the mean standard deviation (blue).

Table 2 shows the VIF values for all models and detailed information regarding geographical regions, PPR outbreak points, elevation, and environmental variables.

**Table 2:** The VIF value of all models and its detailed information of geographical region, PPR occurrence point, elevation, and environment variables.

Models	Köppen type	Natural break	Filtering point	AUC	SD	Environmental layers	VIF
SDM‐1	Af	5 km	8	0.962	0.022	Land cover, soil, Prec11	1.001–1.486
SDM‐2	Am	45 km	12	0.910	0.05	Land cover, soil, Bio14	1.013–1.043
SDM‐3	Aw	5 km	57	0.904	0.02	Population, soil, land cover	1.022–1.083
SDM‐4	Bs	20 km	68	0.919	0.019	Prec1, soil, population, land cover	1.013–1.253
SDM‐5	Bw	20 km	109	0.929	0.014	Population, Prec1, soil	1.002–1.005
SDM‐6	Cf	5 km	5	0.988	0.01	Bio4, soil, land cover, Tmin9	1.017–5.604
SDM‐7	Cs	40 km	60	0.884	0.017	Prec12, population, soil, land cover	1.002–1.308
SDM‐8	Cwa	20 km	5	0.976	0.01	Land cover, soil, Bio6	1.011–1.121
SDM‐9	Cwb	50 km	5	0.936	0.068	Land cover, soil, Prec5	1.001–2.000

The results indicate that high‐risk areas are primarily concentrated in northern and central Africa, consistent with the documented spread trajectory of PPR outbreaks. From the perspective of the origin and diffusion mechanism of the epidemic, PPR first emerged along the Mediterranean coastal region of northern Africa. As the epidemic has not been effectively contained in this area, northern Africa remains a high‐risk zone in the prediction model. Furthermore, considering the trend of PPR spreading southeastward from northern Africa, a large region of medium‐to‐high risk zones has formed in the Middle and Eastern parts of Africa.

From the perspective of environmental constraints, the Sahara Desert region, due to its extreme climate conditions and low population density, combined with an unfavorable meteorological environment for virus survival and transmission, effectively suppresses the spread and diffusion of PPR. As a result, most of the desert area is classified as a low‐risk zone. In contrast, Southern Africa, an area not yet affected by the PPR epidemic, is also considered a low‐risk zone, assuming strict implementation of control and quarantine measures. Under these conditions, the risk of PPR introduction and spread remains relatively low (Figure 6).

Figure 6Risk prediction map based on the MaxEnt model. (a) Prediction of spatial distribution of PPR in Africa. (b) Prediction of potential regional levels of PPR in Africa, the potential regional levels are high, medium, low, and not suitable. The map was made in ArcGIS 10.8 using the resulting rasters produced by MaxEnt.

**Figure:**
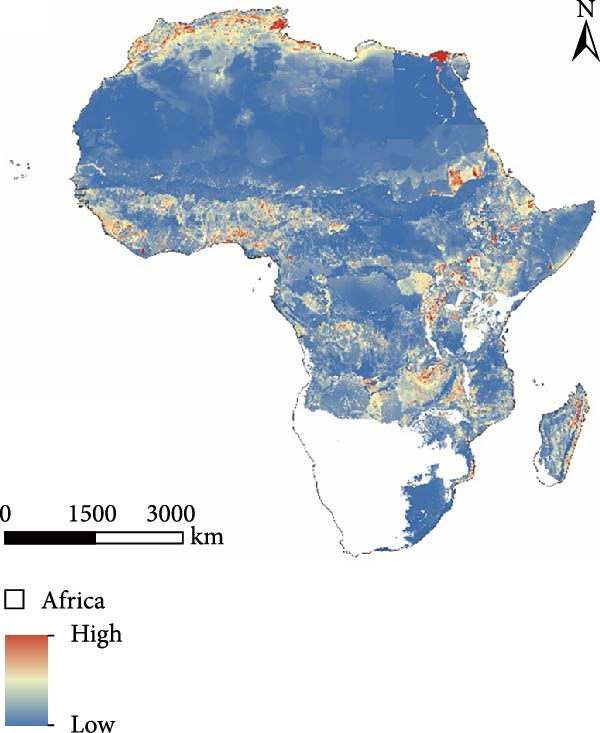
(a)

**Figure:**
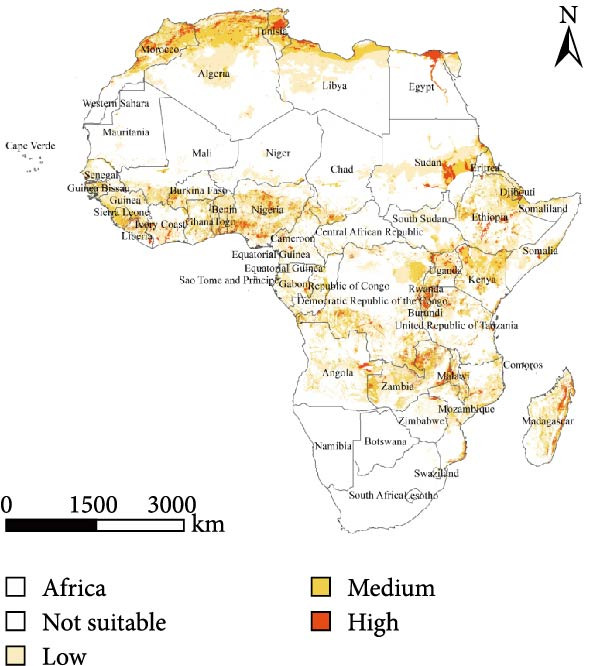
(b)

## 5. Discussion

This study employs GIS to integrate multiple datasets and applies the MaxEnt ecological niche model to identify key drivers of PPR outbreaks across Africa. By integrating historical outbreak records and spatially stratified model outputs, we demonstrate that environmental variables exert significant regulatory control over the survival, transmission, and emergence of the PPRV. Moreover, complex anthropogenic factors—including human population dynamics and livestock movement—further contribute to the spatial diffusion of the disease.

In our study, the PPR‐affected regions of Africa were divided into nine distinct SDMs. Among these, four models identified population density as a key determinant of outbreak occurrence, underscoring its critical role in disease dynamics—a pattern consistent with other directly transmitted infections.

Most African countries remain heavily reliant on agriculture, with livestock production forming a major pillar of rural economies. High population density often correlates with intensified demand for animal products, driving active livestock trade networks. Urban centers and trade hubs—such as Sahelian markets in West Africa—serve as major nodes of animal movement, where viruses can spread rapidly via live animal transactions, shared transport infrastructure, and human contact. For example, Lagos State in Nigeria, characterized by high population density and large‐scale livestock trade, has emerged as a persistent PPR hotspot.

Increased population pressure also intensifies competition for land and grazing resources. As arable and pastoral land becomes scarce, herders are forced to overutilize limited areas, leading to elevated livestock densities and higher contact rates—conditions conducive to viral transmission. Many densely populated informal settlements and rural communities in Africa lack adequate veterinary infrastructure, sanitation, and access to surveillance and vaccination. These gaps delay outbreak detection and response, increasing disease burden. For instance, recurrent PPR outbreaks in densely populated areas of the DRC are most likely linked to low vaccination coverage. Beyond population density, ecological and climatic factors exert substantial influence on the spatial distribution of PPR outbreaks [51]. Among the nine SDMs constructed, six models identified precipitation as a significant predictor, with January rainfall emerging as a key variable in both SDM‐4 and SDM‐5. Notably, both excessive and insufficient annual precipitation were associated with a suppressive effect on PPR occurrence. Instead, outbreak risk was highest under semiarid conditions characterized by distinct wet and dry seasons and annual precipitation ranging between 300–1200 mm [52].

These findings suggest that alternating drought and rainy periods may increase outbreak risk. Drought‐induced forage scarcity can compromise animal nutrition and immunity, increasing susceptibility to infection. Although the onset of the rainy season may temporarily minimize drought stress, it also raises the risk of flooding [53]. After rainfall, the soil often stays moist for some time, creating a favorable environment that helps the virus survive briefly in animal feces and secretions [54]. This short‐term survival can make it easier for the virus to spread indirectly between animals. This hypothesis aligns with outbreak patterns reported in Tanzania, where PPR has been confirmed to circulate widely, particularly in semiarid regions with low relative humidity [52, 55].

Furthermore, seasonal transitions triggered by uneven precipitation—particularly from dry to wet seasons—can induce physiological stress in livestock, further reducing immune defenses and amplifying disease vulnerability.

This study identifies local temperature as a contributing factor in the dynamics of PPR outbreaks, though not as a primary driver. MaxEnt model outputs suggest a dual role of temperature: low temperatures appear to suppress viral transmission, whereas moderate increases—particularly in the range of 15°C–20°C—are associated with elevated outbreak risk. However, the effect of further increases in minimum temperature remains uncertain. We hypothesize that excessively high temperatures may again inhibit viral activity, as evidenced by the consistently low incidence of PPR in the hyper‐arid, high‐temperature zones of the Sahara [56].

These observations suggest a nonlinear relationship between temperature and outbreak probability, with a potential ecological threshold between 10°C and 25°C—consistent with findings from other infectious disease systems [57]. Identification of such thresholds could serve as an early warning indicator for climate‐sensitive PPR emergence.

In addition, seasonal temperature variability may influence host movement patterns, particularly in pastoralist systems. Fluctuations in temperature often influence pastoralist communities to migrate their livestock across regional or national borders in pursuit of adequate water and forage resources. Such transboundary livestock movements elevate the risk of long‐distance transmission of infectious diseases by facilitating contact between diverse herds and exposing animals to novel pathogens within new environments. [58].

The survival and transmission of PPRV are influenced by ecological thresholds of temperature and humidity [57]. Extremes in either temperature or humidity likely reduce viral viability and constrain epidemic potential. For instance, in SDM‐1—representing tropical rainforest zones characterized by annual precipitation exceeding 2000 mm and stable mean temperatures above 26°C—PPR outbreaks were virtually absent. This observation is consistent with the MaxEnt model predictions, underscoring the ecological unsuitability of such hyper‐humid and thermally stable environments for PPRV persistence.

This study show that land cover and soil type emerged as critical factors influencing the spatial dynamics of PPR outbreaks. MaxEnt modeling indicates that outbreak risk increases in areas dominated by urban land, croplands, shrublands, and herbaceous vegetation. Urban areas, characterized by high population density and intensified livestock trade, create environments that facilitate frequent host interactions and enhance viral transmission. Moreover, in many African cities, regulatory oversight of live animal movement and quarantine enforcement remains limited, further facilitating disease spread [59].

Shrublands and grasslands, which form the core of nomadic and seminomadic pastoral systems, support high livestock densities and mobility—conditions that favor direct‐contact transmission. For example, in East Africa’s savannah zones, concentrated grazing activity has been linked to rapid epidemic expansion.

In mixed‐use croplands, the integration of agricultural and livestock activities elevates the potential for inter‐herd contact, especially when livestock are allowed to forage in cultivated fields. Furthermore, agricultural activities, such as the transport of goods and animals, may also serve as vectors for viral dissemination [60]. The frequent outbreaks observed in the Sahel region of West Africa—characterized by semiarid agropastoral landscapes—may reflect this complex land use interface.

Soil type plays a more indirect role by modulating environmental stability. Fertile soils support high‐quality forage, contributing to animal health and resilience, while infertile or sandy soils may limit pasture productivity, indirectly increasing susceptibility to disease due to nutritional stress.

In addition to the outbreak drivers identified by the MaxEnt model, spatial analyses using ArcGIS hotspot mapping and kernel density estimation reveal notable patterns in outbreak frequency and geographic shift. In North Africa, Morocco has recorded the highest number of PPR outbreaks, followed by Algeria and Tunisia. Between 2008 and 2025, the spatial distribution of outbreaks has shown considerable variability and expansion into new geographic areas.

Directional statistical analyses—such as MC shifts, standard deviational ellipses, and LDM—consistently indicate a southeastward spread of PPR across the African continent. This trend aligns with findings from previous studies [61]. The LDM trajectories further demonstrate the virus’s capacity for long‐distance, cross‐regional transmission, likely driven by transhumant pastoralism and cross‐border live animal trade. These patterns show that how livestock mobility—often transcending administrative boundaries—facilitates the sustained spatial propagation of PPR along established trade and migration corridors.

In summary, the spread of PPR across Africa is driven by a complex interplay of environmental, ecological, and socioeconomic factors. Precipitation and temperature influence outbreak risk indirectly by affecting animal health and immunity, while land cover, particularly in urban, pastoral, and agricultural zones, provides geographical pathways for virus transmission. High population density and frequent livestock trade significantly enhance the transmission dynamics within and beyond infected regions.

Effective control strategies must be tailored to regional contexts. For example, promoting drought‐resilient forage species in arid regions, strengthening regulation of live animal movement in densely populated areas, and improving veterinary service capacity at the community level are critical interventions. In high‐risk zones characterized by favorable climatic conditions, high human and livestock densities, but limited geographic extent, outbreaks often follow a satellite‐like pattern—sustained primarily through cross‐regional spillover from adjacent endemic areas due to the limited host population size.

As sheep and goat densities continue to rise across Africa, the potential for PPRV transmission is likely to increase. In light of the observed northwest‐to‐southeast directional spread identified through LDM analysis, enhanced screening of livestock imported from endemic regions is essential. Early detection, prompt reporting of suspected cases, and enhanced border surveillance and quarantine measures are essential, particularly during the transition from the dry to the rainy season in densely populated regions. Furthermore, improved regional coordination and information sharing among neighboring countries will be essential for mitigating transboundary transmission and ensuring the long‐term stability of livestock production systems.

Our analysis of the drivers of PPR outbreaks and the spatial trajectory of its epidemic center provides deeper insight into the disease’s transmission dynamics and highlights the pivotal role of demographic, geographic, and environmental factors. Given the unique transmission dynamics of PPRV, it is imperative to develop scientifically evidence‐based, region‐specific control strategies that address the primary factors driving its spread.

In regions of North and Central Africa where PPR is already endemic, efforts should focus on strengthening grassroots veterinary infrastructure to ensure rapid detection and response. In Southern Africa—where climatic conditions may favor future outbreaks—enhanced quarantine measures and movement surveillance are essential to prevent the southward progression of the epidemic center along the projected trajectory. Timely and well‐targeted implementation of these measures can significantly reduce the overall burden on both human and material resources, while improving the resilience of livestock systems to future incursions.

## Conflicts of Interest

The authors declare no conflicts of interest.

## Funding

No funding was received for this manuscript.

## Supporting Information

Additional supporting information can be found online in the Supporting Information section.

## Supporting information


**Supporting Information** Figure S1: ROC graphs Receiver operating characteristic (ROC) curves from SDM1–9. Figure S2: The response curves for important variables from SDM1–5. Figure S3: The response curves for important variables from SDM 6–9. Table S1: Statistical Chart of Peste des Petits Ruminants Outbreak Frequencies in African Countries. Table S2: Supporting Data on the Peste des petits ruminants Outbreaks in Africa.

## Data Availability

Data are available on request from the authors.
