# Supplementary material for: Analysing Spatiotemporal Characteristics and Estimating the Spatial Distribution of Peste des Petits Ruminants (PPR) in Africa
Source: Transbound Emerg Dis. 2026 Jan 5;2026:9501187. doi: 10.1155/tbed/9501187 (PMC12766275; doi:10.1155/tbed/9501187)
Supplement: Supplementary file 1 — Supporting Information Figure S1: ROC graphs Receiver operating characteristic (ROC) curves from SDM1–9. Figure S2: The response curves for important variables from SDM1–5. Figure S3: The response curves for important variables from SDM 6–9. Table S1: Statistical Chart of Peste des Petits Ruminants Outbreak Frequencies in African Countries. Table S2: Supporting Data on the Peste des petits ruminants Outbreaks in Africa. [file TBED-2026-9501187-s001.docx]

**Supplementary Materials for**

**Analysising spatiotemporal characteristics and estimating the spatial distribution of peste des petits ruminants (PPR) in Africa**

**This file includes:**

**Supplementary methods**

The supplementary introduce the calculation principle of the spatiotemporal analysis method in detail, including Hot Spot analysis, Kernel Density Estimation, Standard Deviational Ellipse, Linear Directional Mean.

**Supplementary figures**

Figure S1. ROC graphs Receiver operating characteristic (ROC) curves from SDM1 to 9

Figure S2. The response curves for important variables from SDM1 to 5

Figure S3. The response curves for important variables from SDM 6 to 9

**Supplementary table files:**

Table S1. Statistical Chart of Peste des Petits Ruminants Outbreak Frequencies in African Countries

Table S2: Supplementary Data on the Peste des petits ruminants Outbreaks in Africa

**Supplementary methods**

Geographic information system, which is composed of data input, management, analysis and presentation, is a comprehensive technology. It can combine a variety of data sources to describe the outbreak and epidemic of animal diseases. In this study, outbreak data were applied to analyze the prevalence of the disease. ArcGIS 10.8(<http://www.esri.com/arcgis>), was used to analyze the temporal and spatial prevalence of animal diseases. Firstly, the longitude and latitude data of disease outbreak sites were collected on emperes-i, FAO and WOAH. The point data (longitude and latitude) in Excel was converted into shape file by ArcGIS software, and the distribution data was displayed by map. Then, Arctoolbox in ArcGIS was applied to the analysis of hot spots analysis, kernel density estimation, standard deviational ellipse, linear directional mean in the prevalence of animal diseases. The principle of the analysis method and the operation of the software were introduced below.

1. Hot Spot analysis

The calculation formula of *G_i_** index is as follows:

$G_{i}^{*}=\frac{\sum_{j=1}^{n} w_{ij}x_{j}-\sum_{j=1}^{n} w_{ij}\bar{x}}{s\sqrt{\frac{\left( n\sum_{j=1}^{n} x_{ij}^{2}-{(\sum_{j=1}^{n} w_{ij})}^{2} \right)}{n-1}}}$ (1)

Where *w_ij_* is the spatial weight between elements *i* and *j*, *x_j_* is the density of element *j*, and n is the number of data points. When *G_i_** value is positive and significant, it presents a hot spot area; when *G_i_** value is negative and significant, it presents a cold spot area.

All operations were completed in ArcGIS 10.8. First, added the base map of Africa with PPR outbreak data, then selected "spatial statistics tool" in system toolboxes, and then selected hot spot analysis (Getis-Ord Gi*) in" cluster distribution mapping "to enter the operation interface. More detailed parameters in the operation interface have been detailed in the method section of the article.

1. Kernel Density Estimation

The principal formula of kernel density estimation with point P as the center is as follows:

$P\left( x,y \right)=\frac{1}{r^{2}}\sum_{i=1}^{n} \left[ \frac{3}{\pi}P_{i}\left( 1-{(\frac{d_{i}}{r})}^{2} \right)^{2} \right]$ for $d_{i}$＜*r* (2)

Where: i=1, ..., n is the input point, P (x, y) is the density prediction value of point (x, y), *p_i_* is the sampling value, *r* is the search radius, *d_i_* is the distance from sampling point *i* to point (x, y).

All operations were completed in ArcGIS 10.8. First, added the base map of Africa with PPR outbreak data, then selected "spatial analyst tools" in system toolboxes, and then selected "nuclear density analysis" in "density analysis" to enter the operation interface. More detailed parameters in the operation interface have been detailed in the method section of the article.

1. Standard Deviational Ellipse

The ellipse calculation formula is as follows:

$\mathrm{SD}E_{a}=\sqrt{\frac{\sum_{i=1}^{n} \left( a_{i}-\bar{A} \right)^{2}}{n}}$ (3)

$\mathrm{SD}E_{b}=\sqrt{\frac{\sum_{i=1}^{n} \left( b_{i}-\bar{B} \right)^{2}}{n}}$ (4)

Where $a_{i}$ and $b_{i}$ is the spatial position coordinate of each element, $\bar{A}$and$\bar{B}$ represent the average center of the arithmetic, and then determine the direction of the ellipse, with the X axis as the standard, the north direction as 0°and the calculation formula of clockwise rotation is as follows:

$\tan\theta=\frac{\sqrt{(\left( \sum_{i=1}^{n} {\alpha_{i}}^{2}-\sum_{i=1}^{n} {\beta_{i}}^{2} \right)^{2}+\left( \sqrt{(\left( \sum_{i=1}^{n} {\alpha_{i}}^{2}-\sum_{i=1}^{n} {\beta_{i}}^{2} \right)^{2}+4\left( \sum_{i=1}^{n} \alpha_{i}\beta_{i} \right)^{2})} \right)}}{2(\sum_{i}^{n} \alpha_{i}\beta_{i})}$ (5)

Where $\alpha_{i}$ and $\beta_{i}$ is the coordinate difference between the average center and $a_{i}$ and $b_{i}$ respectively. Finally, the standard deviation of XY axis is determined. The calculation formula is as follows:

$\sigma_{a}=\sqrt{2}\sqrt{\frac{{(\alpha_{i}\cos\theta-\beta_{i}\sin\theta)}^{2}}{n}}$ (6)

$\sigma_{\beta}=\sqrt{2}\sqrt{\frac{{(\alpha_{i}\sin\theta+\beta_{i}\cos\theta)}^{2}}{n}}$ (7)

All operations were completed in ArcGIS 10.8. First, added the base map of Africa with PPR outbreak data, then selected "spatial statistics tool" in system toolboxes, and then selected "direction distribution (standard deviation ellipse)" in "measuring geographical distribution", and then enter the operation interface. More detailed parameters in the operation interface have been detailed in the method section of the article.

1. Linear Directional Mean

The calculation formula of average value in linear direction is as follows:

$LDM=arc\tan\frac{\sum_{i=1}^{n} \sin\theta_{i}}{\sum_{i=1}^{n} \cos\theta_{i}}$ (8)

Where $\theta_{i}$ is the direction of a group of polyline elements starting from a single source.

All operations were completed in ArcGIS 10.8. The specific operation methods are as follows:

1. added the base map of Africa with PPR outbreak data.
2. use the spatial statistics tool in ArcGIS software to calculate the mean center of the data, determining the central reference point of the data distribution.
3. use the data management tool to merge the calculated mean centers into a unified central dataset, laying the foundation for subsequent analysis.
4. use the feature conversion function to transform the point data into polyline data, changing the data format to meet the LDM analysis requirement for linear features.
5. selected "spatial statistics tool" in system toolboxes, and then selected "linear directional mean" in "measuring geographical distribution", and calculate and generate the direction with arrow representing the overall trend.

**Supplementary figures**

**Figure S1.:** ROC graphs Receiver operating characteristic (ROC) curves from SDM1 to 9

**
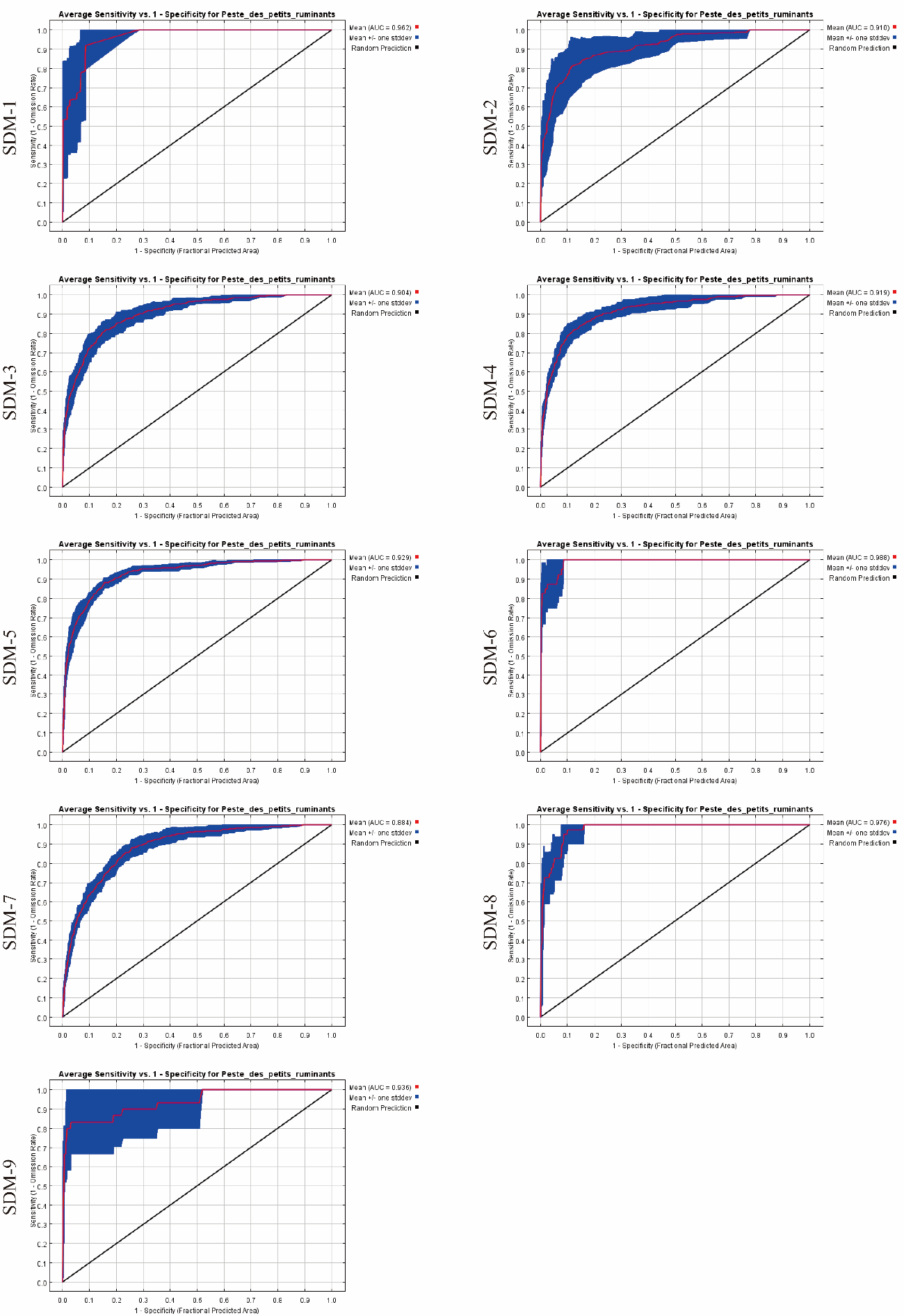
**

**Figure S2:** The response curves for important variables from SDM1 to 5


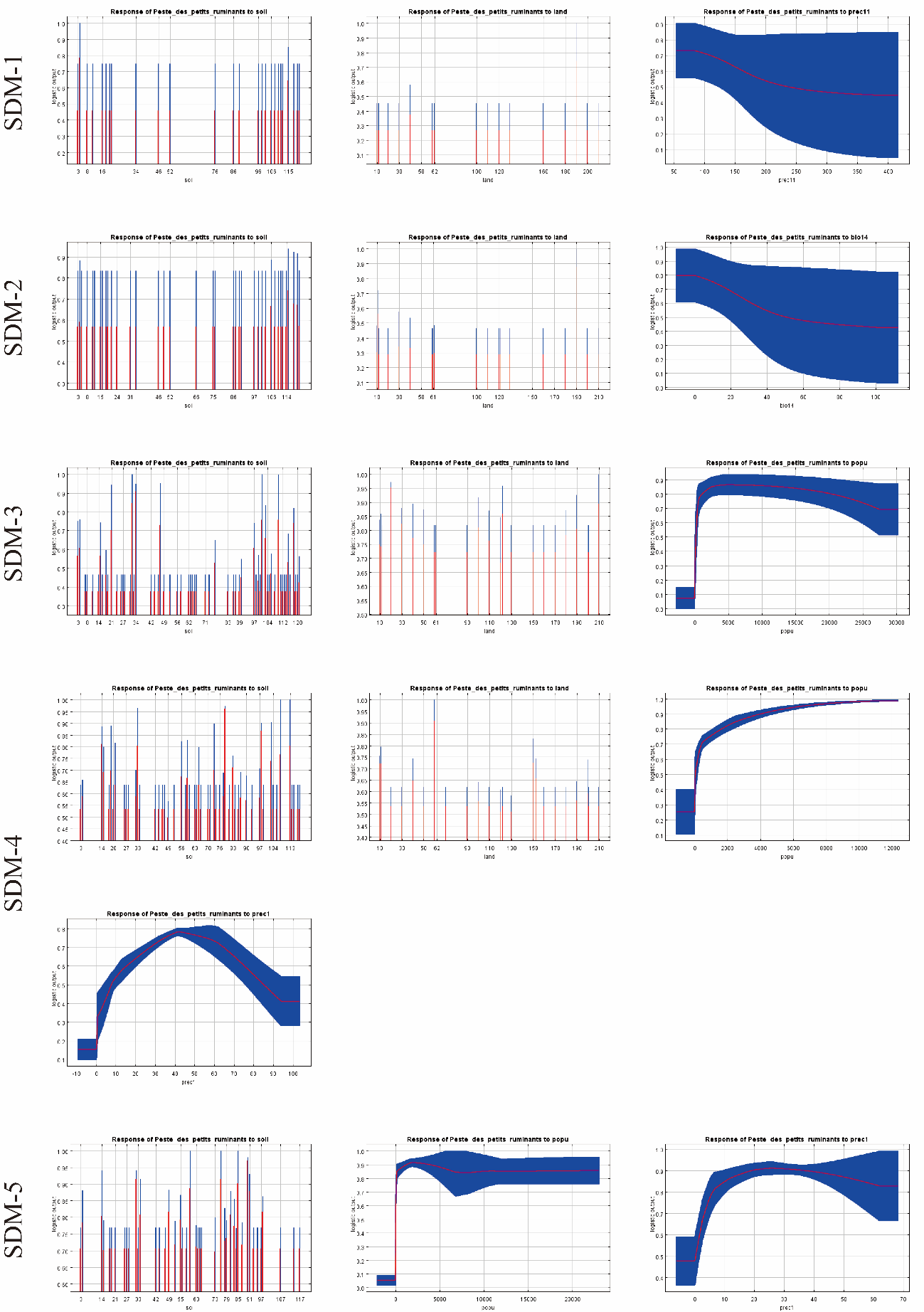


**Figure S3:** The response curves for important variables from SDM 6 to 9

**
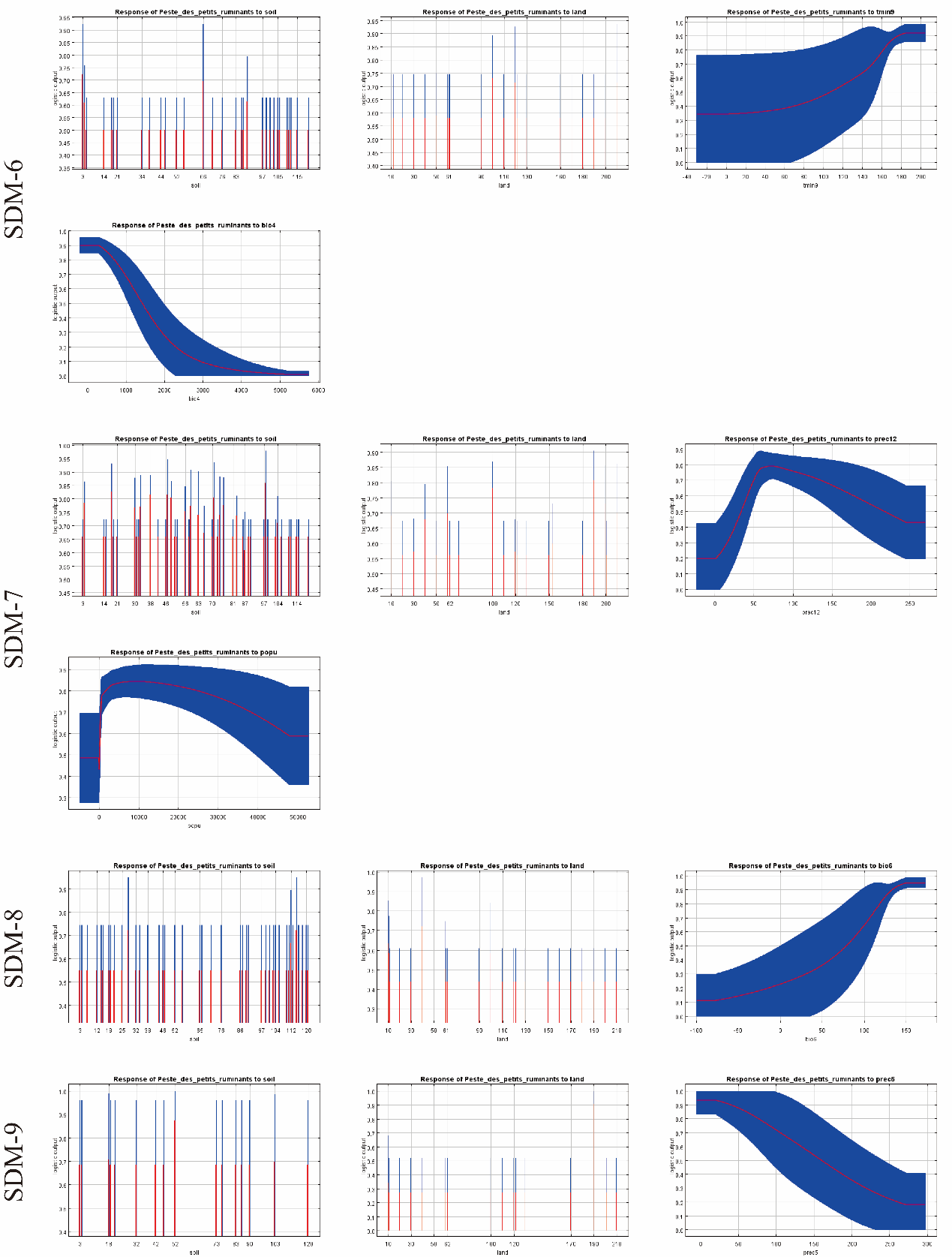
**

**Table S1:** Statistical Chart of Peste des Petits Ruminants Outbreak Frequencies in African Countries

|  | **Number of outbreaks per year** | | | | | | | | | | | | | | | | | | | |
| --- | --- | --- | --- | --- | --- | --- | --- | --- | --- | --- | --- | --- | --- | --- | --- | --- | --- | --- | --- | --- |
| **Country** | **2005** | | **2006** | | **2007** | | **2008** | | **2009** | | **2010** | | **2011** | | **2012** | | **2013** | | **2014** | |
| Algeria | 0 | | 0 | | 0 | | 0 | | 0 | | 0 | | 7 | | 3 | | 4 | | 0 | |
| Angola | 0 | | 0 | | 0 | | 0 | | 0 | | 0 | | 0 | | 1 | | 0 | | 0 | |
| Burkina Faso | 0 | | 0 | | 0 | | 0 | | 0 | | 0 | | 0 | | 0 | | 0 | | 0 | |
| Burundi | 0 | | 0 | | 0 | | 0 | | 0 | | 0 | | 0 | | 0 | | 0 | | 0 | |
| Comoros | 0 | | 0 | | 0 | | 0 | | 0 | | 0 | | 0 | | 5 | | 0 | | 0 | |
| Congo | 1 | | 3 | | 0 | | 0 | | 0 | | 0 | | 0 | | 0 | | 0 | | 0 | |
| Democratic Republic of the Congo | 0 | | 0 | | 0 | | 0 | | 0 | | 0 | | 0 | | 5 | | 0 | | 0 | |
| Egypt | 0 | | 0 | | 0 | | 0 | | 0 | | 1 | | 0 | | 2 | | 2 | | 0 | |
| Gabon | 0 | | 0 | | 0 | | 0 | | 0 | | 0 | | 1 | | 0 | | 0 | | 0 | |
| Kenya | 0 | | 7 | | 3 | | 0 | | 0 | | 0 | | 0 | | 0 | | 3 | | 0 | |
| Liberia | 0 | | 0 | | 0 | | 0 | | 0 | | 0 | | 0 | | 0 | | 0 | | 0 | |
| Libya | 0 | | 0 | | 0 | | 0 | | 0 | | 0 | | 0 | | 0 | | 0 | | 0 | |
| Mali | 2 | | 0 | | 0 | | 0 | | 0 | | 0 | | 0 | | 0 | | 0 | | 0 | |
| Mauritania | 0 | | 0 | | 0 | | 0 | | 0 | | 0 | | 0 | | 0 | | 0 | | 0 | |
| Morocco | 0 | | 0 | | 0 | | 397 | | 0 | | 0 | | 0 | | 5 | | 0 | | 0 | |
| Rwanda | 0 | | 0 | | 0 | | 0 | | 0 | | 0 | | 0 | | 0 | | 0 | | 0 | |
| Senegal | 0 | | 0 | | 0 | | 0 | | 0 | | 0 | | 1 | | 0 | | 0 | | 0 | |
| Sierra Leone | 0 | | 0 | | 0 | | 0 | | 0 | | 0 | | 0 | | 0 | | 0 | | 0 | |
| Tunisia | 0 | | 0 | | 0 | | 0 | | 0 | | 0 | | 5 | | 22 | | 0 | | 0 | |
| Tanzania | 0 | | 0 | | 1 | | 1 | | 2 | | 0 | | 0 | | 0 | | 0 | | 0 | |
| Zambia | 0 | | 0 | | 0 | | 0 | | 0 | | 0 | | 0 | | 0 | | 0 | | 0 | |
| total | 3 | | 10 | | 4 | | 398 | | 2 | | 1 | | 14 | | 43 | | 9 | | 0 | |
|  | | **Number of outbreaks per year** | | | | | | | | | | | | | | | | | | |
| **Country** | | **2015** | | **2016** | | **2017** | | **2018** | | **2019** | | **2020** | | **2021** | | **2022** | | **2023** | | **2024** |
| Algeria | | 0 | | 1 | | 0 | | 26 | | 80 | | 0 | | 1 | | 18 | | 0 | | 0 |
| Angola | | 0 | | 0 | | 0 | | 0 | | 0 | | 0 | | 0 | | 0 | | 0 | | 0 |
| Burkina Faso | | 0 | | 0 | | 0 | | 0 | | 0 | | 0 | | 0 | | 0 | | 0 | | 0 |
| Burundi | | 0 | | 0 | | 0 | | 8 | | 0 | | 0 | | 0 | | 0 | | 0 | | 0 |
| Comoros | | 0 | | 0 | | 0 | | 0 | | 0 | | 0 | | 0 | | 0 | | 0 | | 0 |
| Congo | | 0 | | 0 | | 0 | | 0 | | 0 | | 0 | | 0 | | 0 | | 0 | | 0 |
| Democratic Republic of the Congo | | 0 | | 0 | | 0 | | 0 | | 0 | | 0 | | 1 | | 0 | | 0 | | 0 |
| Egypt | | 0 | | 0 | | 0 | | 0 | | 0 | | 0 | | 0 | | 0 | | 0 | | 0 |
| Gabon | | 0 | | 0 | | 0 | | 0 | | 0 | | 0 | | 0 | | 0 | | 0 | | 1 |
| Kenya | | 0 | | 0 | | 0 | | 0 | | 0 | | 0 | | 0 | | 0 | | 0 | | 0 |
| Liberia | | 5 | | 0 | | 0 | | 0 | | 0 | | 0 | | 0 | | 0 | | 0 | | 0 |
| Libya | | 0 | | 0 | | 0 | | 0 | | 1 | | 5 | | 0 | | 0 | | 0 | | 0 |
| Mali | | 0 | | 0 | | 0 | | 0 | | 0 | | 0 | | 0 | | 0 | | 0 | | 0 |
| Mauritania | | 0 | | 0 | | 0 | | 0 | | 0 | | 0 | | 0 | | 2 | | 0 | | 0 |
| Morocco | | 10 | | 0 | | 0 | | 0 | | 0 | | 3 | | 7 | | 4 | | 0 | | 0 |
| Rwanda | | 0 | | 0 | | 0 | | 0 | | 0 | | 0 | | 0 | | 0 | | 1 | | 0 |
| Senegal | | 0 | | 0 | | 0 | | 0 | | 0 | | 0 | | 0 | | 0 | | 0 | | 0 |
| Sierra Leone | | 0 | | 0 | | 0 | | 7 | | 0 | | 0 | | 0 | | 0 | | 0 | | 0 |
| Tunisia | | 0 | | 24 | | 0 | | 0 | | 0 | | 0 | | 0 | | 0 | | 0 | | 0 |
| Tanzania | | 0 | | 0 | | 0 | | 0 | | 0 | | 0 | | 0 | | 0 | | 0 | | 0 |
| Zambia | | 4 | | 0 | | 0 | | 0 | | 0 | | 0 | | 0 | | 0 | | 0 | | 0 |
| total | | 19 | | 25 | | 0 | | 41 | | 81 | | 8 | | 9 | | 24 | | 1 | | 1 |

* All country names are standardized in accordance with ISO 3166-1

* The data were collected from WOAH and EMPRES-i

**Table S2:** Supplementary Data on the Peste des petits ruminants Outbreaks in Africa

| **Country** | **Longitude** | **Latitude** | **Reference** |
| --- | --- | --- | --- |
| Algeria | 6.471658 | 36.283187 | First Evidence of Peste des Petits Ruminants (PPR) Virus Circulation in Algeria (Sahrawi Territories): Outbreak Investigation and Virus Lineage Identification |
|  | -12.9833 | 27.167 | First Evidence of Peste des Petits Ruminants (PPR) Virus Circulation in Algeria (Sahrawi Territories): Outbreak Investigation and Virus Lineage Identification |
|  | 3.6733 | 32.4908 | First serological and molecular evidence of PPRV occurrence in Ghardaïa district, center of Algeria |
| Egypt | 31.1841 | 30.4625 | An outbreak of peste des petits ruminants in migratory flocks of sheep and goats in Egypt in 2006 |
|  | 30.9129 | 31.2903 | An outbreak of peste des petits ruminants in migratory flocks of sheep and goats in Egypt in 2006 |
|  | 31.193945 | 30.356046 | An outbreak of peste des petits ruminants in migratory flocks of sheep and goats in Egypt in 2006 |
|  | 32.274444 | 30.586111 | Emergence of peste des petits ruminants virus lineage IV in Ismailia Province, Egypt |
|  | 9.183333 | 36.733333 | Emergence of peste des petits ruminants virus lineage IV in Ismailia Province, Egypt |
|  | 9.1833 | 36.7333 | Emergence of peste des petits ruminants virus lineage IV in Ismailia Province, Egypt |
|  | 9.9 | 37.25 | Emergence of peste des petits ruminants virus lineage IV in Ismailia Province, Egypt |
|  | 8.8 | 34.4167 | Emergence of peste des petits ruminants virus lineage IV in Ismailia Province, Egypt |
|  | 8.7417 | 36.6925 | Emergence of peste des petits ruminants virus lineage IV in Ismailia Province, Egypt |
|  | 9.8667 | 35.675 | Emergence of peste des petits ruminants virus lineage IV in Ismailia Province, Egypt |
|  | 8.6944 | 36.0433 | Emergence of peste des petits ruminants virus lineage IV in Ismailia Province, Egypt |
|  | 9.85 | 36.85 | Emergence of peste des petits ruminants virus lineage IV in Ismailia Province, Egypt |
|  | 10.4747 | 33.3558 | Emergence of peste des petits ruminants virus lineage IV in Ismailia Province, Egypt |
|  | 10.8203 | 35.7744 | Emergence of peste des petits ruminants virus lineage IV in Ismailia Province, Egypt |
|  | 10.7347 | 36.4597 | Emergence of peste des petits ruminants virus lineage IV in Ismailia Province, Egypt |
|  | 10.7603 | 34.7447 | Emergence of peste des petits ruminants virus lineage IV in Ismailia Province, Egypt |
|  | 9.49 | 35.0167 | Emergence of peste des petits ruminants virus lineage IV in Ismailia Province, Egypt |
|  | 9.3295 | 36.0509 | Emergence of peste des petits ruminants virus lineage IV in Ismailia Province, Egypt |
|  | 9.943 | 31.9995 | Emergence of peste des petits ruminants virus lineage IV in Ismailia Province, Egypt |
|  | 10.1472 | 36.4025 | Emergence of peste des petits ruminants virus lineage IV in Ismailia Province, Egypt |
|  | 10.0955 | 36.8548 | An outbreak of peste des petits ruminants in migratory flocks of sheep and goats in Egypt in 2006 |
|  | 32.34375 | 30.70539 | Prevalence and molecular characterization of peste des petits ruminants virus from Ismailia and Suez, Northeastern Egypt, 2014-2016 |
|  | 32.25 | 29.97 | Prevalence and molecular characterization of peste des petits ruminants virus from Ismailia and Suez, Northeastern Egypt, 2014-2016 |
| Sierra Leone | -12.433333 | 8.160556 | Complete Genome Sequence of a Lineage II Peste des Petits Ruminants Virus from Sierra Leone |
|  | -12.0491 | 8.8847 | Genetic Characterization of Peste des Petits  Ruminants Virus, Sierra Leone |
| Côte d’Ivoire | 2.95 | 36.766667 | Peste des petits ruminants (PPR): A neglected tropical disease in Maghreb region of North Africa and its threat to Europe |
| Tunisia | 9.5 | 35.0333 | Peste des Petits Ruminants Virus, Tunisia, 2012–2013 |
|  | 9.8632 | 35.6066 | Peste des Petits Ruminants Virus, Tunisia, 2012–2013 |
| Burundi | 29.9 | -3.433333 | Detection and molecular characterization of Peste des Petits Ruminants virus from outbreaks in Burundi, December 2017–January 2018 |
| Ethiopia | 38.74689 | 9.02497 | Peste des petits ruminants in Ethiopian goats |
|  | 38.0477 | 11.5826 | Peste des Petits Ruminants (PPR) in Ethiopia: Analysis of a national serological survey |
|  | 34.65 | 7.65 | Serological investigation of Peste des Petits Ruminants (PPR) in small ruminants managed under pastoral and agro-pastoral systems in Ethiopia |
|  | 34.5833 | 8.25 | Serological investigation of Peste des Petits Ruminants (PPR) in small ruminants managed under pastoral and agro-pastoral systems in Ethiopia |
|  | 39.5 | 7.75 | Serological Investigation of Peste Des Petits Ruminants in East Shewa and Arsi Zones, Oromia Region, Ethiopia |
|  | 38.7 | 7.866667 | Serological Investigation of Peste Des Petits Ruminants in East Shewa and Arsi Zones, Oromia Region, Ethiopia |
|  | 38.3 | 8.783333 | Serological Investigation of Peste Des Petits Ruminants in East Shewa and Arsi Zones, Oromia Region, Ethiopia |
|  | 37.75 | 6.25 | Sero-epidemiological study of peste des petits ruminants (PPR) in sheep and goats under different production systems in South Omo, southern Ethiopia |
|  | 36.68 | 5.93 | Sero-epidemiological study of peste des petits ruminants (PPR) in sheep and goats under different production systems in South Omo, southern Ethiopia |
|  | 39.48 | 14.25 | Sero-epidemiological study of peste des petits ruminants (PPR) in sheep and goats under different production systems in South Omo, southern Ethiopia |
|  | 40.3333 | 12.25 | Molecular detection and phylogenetic analysis of Peste des petits ruminants virus circulating in small ruminants in eastern Amhara region, Ethiopia |
|  | 39.0333 | 11.5 | Molecular detection and phylogenetic analysis of Peste des petits ruminants virus circulating in small ruminants in eastern Amhara region, Ethiopia |
|  | 37.6667 | 11.1833 | Molecular detection and phylogenetic analysis of Peste des petits ruminants virus circulating in small ruminants in eastern Amhara region, Ethiopia |
|  | 38.500278 | 9.215639 | Molecular epidemiological update of Peste des Petits Ruminants virus (PPRV) in Ethiopia |
|  | 39.532619 | 9.679539 | Molecular epidemiological update of Peste des Petits Ruminants virus (PPRV) in Ethiopia |
|  | 35.533333 | 10.766667 | Molecular epidemiological update of Peste des Petits Ruminants virus (PPRV) in Ethiopia |
|  | 41.866667 | 9.6 | Molecular epidemiological update of Peste des Petits Ruminants virus (PPRV) in Ethiopia |
|  | 39.466667 | 13.483333 | Molecular epidemiological update of Peste des Petits Ruminants virus (PPRV) in Ethiopia |
|  | 42.8 | 9.35 | Molecular epidemiological update of Peste des Petits Ruminants virus (PPRV) in Ethiopia |
|  | 41.008611 | 11.792222 | Molecular epidemiological update of Peste des Petits Ruminants virus (PPRV) in Ethiopia |
|  | 40.016667 | 11.183333 | Molecular epidemiological update of Peste des Petits Ruminants virus (PPRV) in Ethiopia |
|  | 37.1 | 9.566667 | Seroepidemiology of Peste des Petits ruminants in sheep and goats in the selected district of Horu Guduru Zone, Western Ethiopia |
|  | 37.333333 | 9.166667 | Seroepidemiology of Peste des Petits ruminants in sheep and goats in the selected district of Horu Guduru Zone, Western Ethiopia |
|  | 38.75 | 4.733333 | Sero-epidemiology of peste des petits ruminants in Oromia and Afar regional states of Ethiopia |
|  | 39.45 | 3.5 | Sero-epidemiology of peste des petits ruminants in Oromia and Afar regional states of Ethiopia |
|  | 37.416667 | 4.833333 | Sero-epidemiology of peste des petits ruminants in Oromia and Afar regional states of Ethiopia |
|  | 38.083333 | 4.883333 | Sero-epidemiology of peste des petits ruminants in Oromia and Afar regional states of Ethiopia |
|  | 35.40416 | 15.2578 | Global ecological niche modelling of current and future distribution of peste des petits ruminants virus (PPRv) with an ensemble modelling algorithm |
| Benin | 2.716667 | 9.433333 | Peste Des Petits Ruminants in Benin: Persistence of a Single Virus Genotype in the Country for Over 42 Years |
|  | 2.566667 | 9.2 | Peste Des Petits Ruminants in Benin: Persistence of a Single Virus Genotype in the Country for Over 42 Years |
|  | 2.65 | 9.583333 | Peste Des Petits Ruminants in Benin: Persistence of a Single Virus Genotype in the Country for Over 42 Years |
| Burkina Faso | −17.41 | 14.74 | Comparative evolutionary analyses of peste des petits ruminants virus genetic lineages |
|  | −16.48 | 14.09 | Comparative evolutionary analyses of peste des petits ruminants virus genetic lineages |
|  | −12.20 | 112.55 | Comparative evolutionary analyses of peste des petits ruminants virus genetic lineages |
|  | −13.96 | 12.76 | Comparative evolutionary analyses of peste des petits ruminants virus genetic lineages |
|  | −17.01 | 14.52 | Comparative evolutionary analyses of peste des petits ruminants virus genetic lineages |
|  | −17.04 | 14.46 | Comparative evolutionary analyses of peste des petits ruminants virus genetic lineages |
|  | -16.84 | 14.18 | Comparative evolutionary analyses of peste des petits ruminants virus genetic lineages |
|  | -15.25 | 10.69 | Comparative evolutionary analyses of peste des petits ruminants virus genetic lineages |
|  | -8 | 12.64 | Comparative evolutionary analyses of peste des petits ruminants virus genetic lineages |
|  | -11.16 | 12.67 | Comparative evolutionary analyses of peste des petits ruminants virus genetic lineages |
|  | -6.9 | 11.08 | Comparative evolutionary analyses of peste des petits ruminants virus genetic lineages |
|  | -6.63 | 11.06 | Comparative evolutionary analyses of peste des petits ruminants virus genetic lineages |
|  | -8.17 | 11.13 | Comparative evolutionary analyses of peste des petits ruminants virus genetic lineages |
|  | -8.15 | 11.18 | Comparative evolutionary analyses of peste des petits ruminants virus genetic lineages |
|  | -9.8 | 12.59 | Comparative evolutionary analyses of peste des petits ruminants virus genetic lineages |
|  | -15.32 | 17.52 | Comparative evolutionary analyses of peste des petits ruminants virus genetic lineages |
|  | -1.56 | 12.24 | Comparative evolutionary analyses of peste des petits ruminants virus genetic lineages |
|  | -0.02 | 5.55 | Comparative evolutionary analyses of peste des petits ruminants virus genetic lineages |
|  | -1.39 | 5.1 | Comparative evolutionary analyses of peste des petits ruminants virus genetic lineages |
|  | 33.6 | 13.55 | Comparative evolutionary analyses of peste des petits ruminants virus genetic lineages |
|  | 88.36 | 22.57 | Comparative evolutionary analyses of peste des petits ruminants virus genetic lineages |
|  | -1.8122 | 12.6076 | Molecular Epidemiology of Peste Des Petits Ruminants Virus inWest Africa: Is Lineage IV Replacing Lineage II in Burkina Faso? |
|  | -4.4308 | 10.2975 | Molecular Epidemiology of Peste Des Petits Ruminants Virus inWest Africa: Is Lineage IV Replacing Lineage II in Burkina Faso? |
|  | -5.5333 | 9.7833 | Molecular Epidemiology of Peste Des Petits Ruminants Virus inWest Africa: Is Lineage IV Replacing Lineage II in Burkina Faso? |
|  | -4.0333 | 12.4833 | Molecular Epidemiology of Peste Des Petits Ruminants Virus inWest Africa: Is Lineage IV Replacing Lineage II in Burkina Faso? |
| Eritrea | 15.32278 | 38.925 | Investigating peste des petits ruminants (PPR) in naturally infected goats and sheep in Anseba Region, Eritrea, by reverse transcription polymerase chain reaction (RT-PCR) |
|  | 14.621281 | 39.194981 | Investigating peste des petits ruminants (PPR) in naturally infected goats and sheep in Anseba Region, Eritrea, by reverse transcription polymerase chain reaction (RT-PCR) |
| Democratic Republic of the Congo | 29.225278 | -1.693333 | Retrospective Characterization of Initial Peste des petits ruminants Outbreaks (2008–2012) in the Democratic Republic of the Congo |
|  | 34.25 | -2.133333 | Retrospective Characterization of Initial Peste des petits ruminants Outbreaks (2008–2012) in the Democratic Republic of the Congo |
|  | 15.404444 | 4.34 | Retrospective Characterization of Initial Peste des petits ruminants Outbreaks (2008–2012) in the Democratic Republic of the Congo |
|  | 15.459167 | 4.391111 | Retrospective Characterization of Initial Peste des petits ruminants Outbreaks (2008–2012) in the Democratic Republic of the Congo |
|  | 26.083333 | -2.116667 | Retrospective Characterization of Initial Peste des petits ruminants Outbreaks (2008–2012) in the Democratic Republic of the Congo |
|  | 28.966667 | -2.116667 | Retrospective Characterization of Initial Peste des petits ruminants Outbreaks (2008–2012) in the Democratic Republic of the Congo |
|  | 28.7 | -2.116667 | Retrospective Characterization of Initial Peste des petits ruminants Outbreaks (2008–2012) in the Democratic Republic of the Congo |
|  | 29.34605 | 1.48247 | Retrospective Characterization of Initial Peste des petits ruminants Outbreaks (2008–2012) in the Democratic Republic of the Congo |
|  | 28.8167 | -1.4 | Retrospective Characterization of Initial Peste des petits ruminants Outbreaks (2008–2012) in the Democratic Republic of the Congo |
|  | 28.05 | 1.4167 | Retrospective Characterization of Initial Peste des petits ruminants Outbreaks (2008–2012) in the Democratic Republic of the Congo |
|  | 13.45 | -5.816667 | Retrospective Characterization of Initial Peste des petits ruminants Outbreaks (2008–2012) in the Democratic Republic of the Congo |
|  | 16.8 | -3.7667 | Retrospective Characterization of Initial Peste des petits ruminants Outbreaks (2008–2012) in the Democratic Republic of the Congo |
|  | 18.85 | -5.041 | Retrospective Characterization of Initial Peste des petits ruminants Outbreaks (2008–2012) in the Democratic Republic of the Congo |
| Mauritania | -12.70931 | 22.67662 | Molecular characterization of peste des petits ruminants virus and Mycoplasma capricolum subsp. capripneumoniae in small ruminants in northern Mauritania, 2023 |
|  | -12.4729 | 22.7374 | Molecular characterization of peste des petits ruminants virus and Mycoplasma capricolum subsp. capripneumoniae in small ruminants in northern Mauritania, 2023 |
|  | -10.95 | 21 | Peste des Petits Ruminants Virus, Mauritania |
| Gabon | 14.13757 | -0.6585 | Molecular typing of PPRV strains detected during an outbreak in sheep and goats in south-eastern Gabon in 2011 |
|  | 7.6244 | 4.6058 | Molecular typing of PPRV strains detected during an outbreak in sheep and goats in south-eastern Gabon in 2011 |
| Comoros | 43.350734 | -11.692104 | Impact and Epidemiological Investigations into the Incursion and Spread of Peste des Petits Ruminants in the Comoros Archipelago: An Increased Threat to Surrounding Islands |
| Kenya | 34.84978 | 3.64416 | Detection and Genome Analysis of a Lineage III Peste Des Petits Ruminants Virus in Kenya in 2011 |
| Liberia | 8.981389 | 7.2375 | First report and characterization of peste des petits ruminants virus in Liberia, West Africa |
| Nigeria | 3.8525 | 7.400833 | Field evaluation and confirmation of acute peste des petits ruminant outbreak in a flock of West African dwarf goats in Ibadan, Nigeria |
|  | 8.5134 | 11.7356 | Field evaluation and confirmation of acute peste des petits ruminant outbreak in a flock of West African dwarf goats in Ibadan, Nigeria |
| Senegal | 14.8082 | 14.22245 | First Report of the Emergence of Peste des Petits Ruminants Lineage IV Virus in Senegal |
| Sudan | 36.381524 | 15.374091 | Epidemiology and molecular  characterization of re-emerged virulent  strains of Peste des Petits Ruminants virus  among sheep in Kassala State, Eastern Sudan |
|  | 36.355586 | 15.470446 | Epidemiology and molecular  characterization of re-emerged virulent  strains of Peste des Petits Ruminants virus  among sheep in Kassala State, Eastern Sudan |
|  | 36.269701 | 15.10806 | Epidemiology and molecular  characterization of re-emerged virulent  strains of Peste des Petits Ruminants virus  among sheep in Kassala State, Eastern Sudan |
|  | 36.22225 | 15.50405 | Epidemiology and molecular  characterization of re-emerged virulent  strains of Peste des Petits Ruminants virus  among sheep in Kassala State, Eastern Sudan |
|  | 35.94391 | 15.5547 | Epidemiology and molecular  characterization of re-emerged virulent  strains of Peste des Petits Ruminants virus  among sheep in Kassala State, Eastern Sudan |
|  | 36.045371 | 15.064989 | Epidemiology and molecular  characterization of re-emerged virulent  strains of Peste des Petits Ruminants virus  among sheep in Kassala State, Eastern Sudan |
|  | 36.15894 | 15.32819 | Epidemiology and molecular  characterization of re-emerged virulent  strains of Peste des Petits Ruminants virus  among sheep in Kassala State, Eastern Sudan |
|  | 36.16069 | 15.26885 | Epidemiology and molecular  characterization of re-emerged virulent  strains of Peste des Petits Ruminants virus  among sheep in Kassala State, Eastern Sudan |
|  | 36.18436 | 15.47355 | Epidemiology and molecular  characterization of re-emerged virulent  strains of Peste des Petits Ruminants virus  among sheep in Kassala State, Eastern Sudan |
|  | 5.1595 | 6.9111 | Epidemiology and molecular characterization of re-emerged virulent strains of Peste des Petits Ruminants virus among sheep in Kassala State, Eastern Sudan |
|  | 32.53241 | 15.505 | First detection and genetic characterization of peste des petits ruminants virus from dorcas gazelles “Gazella dorcas” in the Sudan, 2016-2017 |
|  | 32.5262 | 15.6031 | First detection and genetic characterization of peste des petits ruminants virus from dorcas gazelles “Gazella dorcas” in the Sudan, 2016-2017 |
|  | 33.449722 | 13.274167 | Peste des petits ruminants outbreaks in White Nile State, Sudan |
|  | 32.32 | 15.6 | Asian Lineage of Peste des Petits Ruminants Virus, Africa |
|  | 35.7365 | 15.8983 | Asian Lineage of Peste des Petits Ruminants Virus, Africa |
|  | 33.4 | 14.933333 | Detection of peste des petits ruminants virus in pneumonic lungs from clinically apparently healthy camels slaughtered at Tambul slaughterhouse, Central Sudan |
|  | 24.4284 | 10.8743 | Current situation of Peste des petits ruminants (PPR) in the Sudan |
|  | 32.7852 | 15.8443 | Current situation of Peste des petits ruminants (PPR) in the Sudan |
|  | 33.2813 | 14.6125 | Current situation of Peste des petits ruminants (PPR) in the Sudan |
|  | 37.036111 | 14.036111 | Current situation of Peste des petits ruminants (PPR) in the Sudan |
|  | 30.4833 | 19.17 | Sero-prevalence of peste des petits ruminants virus antibodies in sheep and goats from the Sudan, 2016–2017 |
|  | 33.4333 | 14.4 | Sero-prevalence of peste des petits ruminants virus antibodies in sheep and goats from the Sudan, 2016–2017 |
|  | 32.5333 | 15.6333 | Sero-prevalence of peste des petits ruminants virus antibodies in sheep and goats from the Sudan, 2016–2017 |
|  | 31.3667 | 17.9167 | Sero-prevalence of peste des petits ruminants virus antibodies in sheep and goats from the Sudan, 2016–2017 |
|  | 33.9333 | 16.9333 | Sero-prevalence of peste des petits ruminants virus antibodies in sheep and goats from the Sudan, 2016–2017 |
| Tanzania | 37.5333 | -6.43333 | Preliminary investigation on presence of peste des petits ruminants in Dakawa, Mvomero district, Morogoro region, Tanzania |
|  | 34.8333 | -2.3308 | Spillover of Peste des petits ruminants virus from domestic to wild ruminants in the Serengeti ecosystem, Tanzania |
| Uganda | 34.67 | 2.53 | Peste des Petits Ruminants serological survey in Karamoja sub region of Uganda by competitive ELISA |
|  | 34.7214 | 1.8522 | Peste des Petits Ruminants serological survey in Karamoja sub region of Uganda by competitive ELISA |
|  | 33.6595 | 2.7067 | Peste des Petits Ruminants serological survey in Karamoja sub region of Uganda by competitive ELISA |
|  | 34.1167 | 3.5167 | Peste des Petits Ruminants serological survey in Karamoja sub region of Uganda by competitive ELISA |

* All country names are standardized in accordance with ISO 3166-1
